# Structural genomic variation in the inbred Scandinavian wolf population contributes to the realized genetic load but is positively affected by immigration

**DOI:** 10.1111/eva.13652

**Published:** 2024-02-07

**Authors:** Linnéa Smeds, Lars S. A. Huson, Hans Ellegren

**Affiliations:** ^1^ Department of Ecology and Genetics, Evolutionary Biology Uppsala University Uppsala Sweden

**Keywords:** *Canis lupus*, conservation genetics, genetic load, genetic rescue, structural variants

## Abstract

When populations decrease in size and may become isolated, genomic erosion by loss of diversity from genetic drift and accumulation of deleterious mutations is likely an inevitable consequence. In such cases, immigration (genetic rescue) is necessary to restore levels of genetic diversity and counteract inbreeding depression. Recent work in conservation genomics has studied these processes focusing on the genetic diversity of single nucleotide polymorphisms. In contrast, our knowledge about structural genomic variation (insertions, deletions, duplications and inversions) in endangered species is limited. We analysed whole‐genome, short‐read sequences from 212 wolves from the inbred Scandinavian population and from neighbouring populations in Finland and Russia, and detected >35,000 structural variants (SVs) after stringent quality and genotype frequency filtering; >26,000 high‐confidence variants remained after manual curation. The majority of variants were shorter than 1 kb, with a distinct peak in the length distribution of deletions at 190 bp, corresponding to insertion events of SINE/tRNA‐Lys elements. The site frequency spectrum of SVs in protein‐coding regions was significantly shifted towards rare alleles compared to putatively neutral variants, consistent with purifying selection. The realized genetic load of SVs in protein‐coding regions increased with inbreeding levels in the Scandinavian population, but immigration provided a genetic rescue effect by lowering the load and reintroducing ancestral alleles at loci fixed for derived SVs. Our study shows that structural variation comprises a common type of in part deleterious mutations in endangered species and that establishing gene flow is necessary to mitigate the negative consequences of loss of diversity.

## 1 INTRODUCTION

The rampaging Anthropocene has put myriad species at risk and necessitated massive conservation efforts. It has also brought conservation genetics into sharp focus because genetic diversity is an essential component of both the short‐term and long‐term survival of populations (Supple & Shapiro, 2018). In the last few decades, new sequencing technologies and the development of bioinformatic methods have opened up new possibilities in the field of conservation genomics. Assessing extinction threat using genomic data is challenging (Schmidt et al., 2023), but there is a growing appreciation that the genetic health of species or populations should be taken into account, including when determining red list status (Brüniche‐Olsen et al., 2018; Frankham et al., 2014; Garner et al., 2020; Petit‐Marty et al., 2021; Schmidt et al., 2023; van Oosterhout, 2021; Willoughby et al., 2015).

Small and isolated populations can be threatened by genomic erosion (Bosse & van Loon, 2022; Frankel & Soulé, 1981; Soulé, 1987; Wright, 1931). Specifically, variation in a small gene pool can be depleted due to genetic drift – the random loss of alleles – and accumulate deleterious mutations that will exacerbate genetic load (Willi et al., 2022). This is likely to lead to a reduction in fitness and successively smaller population size over time, a phenomenon referred to as “the extinction vortex” (Gilpin & Soulé, 1986). Genetic rescue is intended to reverse this process – when new (or previously lost) alleles enter the population and thereby alleviate inbreeding, increase genetic diversity (Fitzpatrick et al., 2020; Hedrick, 2005; Hedrick et al., 2011; Robinson et al., 2021; Tallmon et al., 2004), and increase population fitness beyond what is expected from the demographic contribution of immigrants (Whiteley et al., 2015). Gene flow and immigration are the biological processes that transfer alleles between populations and potentially provide genetic rescue effects. By now, there are many examples of directly observed or assumed fitness benefits of genetic rescue from human‐mediated conservation actions (assisted gene flow) or from naturally occurring immigration (Whiteley et al., 2015). For example, translocations programs in the adder (Madsen et al., 1999), Florida panther (Pimm et al., 2006) and mountain pygmy possum (Weeks et al., 2017) had positive effects on population size or fitness. Similar effects have been seen after natural immigration to inbred populations of Arctic foxes (Hasselgren et al., 2018) and grey wolves (Adams et al., 2011), including to the highly inbred Scandinavian wolf population (Åkesson et al., 2016; Vilà et al., 2003), which is the focus of this study.

After wolves were extirpated from Scandinavia in the 1960s, the current Scandinavian wolf population was founded in the 1980s by the arrival of a single reproducing pair (Wabakken et al., 2001). A single immigrant male (the “third founder”) successfully reproduced in 1991, which sparked the transition from a single reproducing pack to an expanding population (Liberg et al., 2005) and initially led to increased heterozygosity (Vilà et al., 2003) and lower inbreeding levels (Åkesson & Svensson, 2022). However, in the absence of further immigration, the population suffered from inbreeding depression (Liberg et al., 2005). In 2008, two new immigrant males bred within the Scandinavian population, and their offspring had higher pairing and reproductive success than inbred individuals from the same period (Åkesson et al., 2016). More recent studies of the population using whole‐genome SNP data have documented a loss of both genotypic and haplotypic diversity over time (Smeds & Ellegren, 2023; Viluma et al., 2022), with 10%–24% of the haplotypes the diploid genome of the three founders being lost after 20 years of drift. Inbreeding has resulted in huge arrays of runs of homozygosity, extending over whole chromosomes in the most inbred individuals (Kardos et al., 2018). It has been previously found that the realized genetic load from deleterious non‐synonymous mutations increased with number of inbreeding generations was balanced by occasional immigration, but then again tended to increase (Smeds & Ellegren, 2023).

Structural variation is a rather heterogenous type of allelic differences in DNA content that either change the total amount of DNA (insertions, deletions or duplications) or change the sequence of DNA (translocations, inversions and fission/fusions). They have been well characterized in the human genome (Alkan et al., 2011), with extensive catalogues of standing structural variation (SV) being available (Collins et al., 2020; Pang et al., 2010). However, this is not the case in natural populations for species of ecological or evolutionary interest, especially not in a population genomic context. But structural variation can have critical consequences for adaptation and speciation, as is evident from the pronounced phenotypic effect of large inversions, representing “supergenes” (Berdan et al., 2021; Funk et al., 2021; Jay et al., 2018; Joron et al., 2011; Kim et al., 2017; Knief et al., 2017; Küpper et al., 2016; Lamichhaney et al., 2016; Merritt et al., 2020). The fact that comparatively little attention has yet been paid to large‐scale patterns of structural variation in non‐human population genetics is somewhat paradoxical given that this type of variation has been known for a century (Creighton & McClintock, 1931; Sturtevant, 1921). This paradox has an interesting historical twist since it relates to methodological developments and limitations both now and 100 years ago. In the early 20th century, cytometry and linkage analyses provided for the first time a means to explore how genetic diversity was organized by the detection of inversions; however, little was known about the genetic material itself, let alone the character of diversity other than what could be seen in the microscope or by laboratory crosses. Now, bioinformatics and new sequencing technologies offer unprecedented possibilities to call genetic variation at the level of individual bases of DNA, but detecting and genotyping inversions and other types of structural variation continues to be more challenging.

So far, most genomic studies of endangered species have focused on single nucleotide polymorphism (SNP) data. At the same time, SVs have largely been ignored, although, attempts have been made to integrate structural variation in large‐scale evolutionary studies of natural populations (Mérot et al., 2020; Weissensteiner et al., 2020) as well as endangered populations (Wold et al., 2023). Here we aim to extend previous work on inbreeding, genetic load and genetic rescue in the Scandinavian wolf population by focusing on SVs as an important category of potentially deleterious mutations. We show that inbreeding increases the realized genetic load contributed by SVs and that immigration provides a genetic rescue effect by reducing the load and reintroducing lost ancestral alleles.

## 2 METHODS

### 2.1 Data acquisition

We gathered short‐read Illumina data from 212 grey wolves (103 Scandinavian, 95 Finnish and 14 Russian Karelian) reported in previous studies (Kardos et al., 2018; Smeds et al., 2019, 2021; Viluma et al., 2022). The animals had been sampled in connection with legal harvesting (or vehicle collisions) or population monitoring (see Åkesson et al. (2022) for ethical permits). The Scandinavian sample set included the female founder of the population and 11 immigrants that were sampled in Scandinavia but originated from the Finnish/Russian population. Seventy‐six individuals descended only from the first three founders of the population; they are here referred to as the “original population” before new immigration events. The reads were mapped to the dog reference genome CanFam3.1 (Lindblad‐Toh et al., 2005) using Burrows‐Wheeler Aligner (bwa) (Li & Durbin, 2009), sorted using samtools (Danecek et al., 2021), and deduplicated with picard (http://broadinstitute.github.io/picard/); for a detailed description see (Kardos et al., 2018). The data were genotyped for SNPs (Smeds et al., 2021) using gatk (Van der Auwera et al., 2013). A list of all samples with accession numbers and metadata is found in Table S1.

### 2.2 Calling structural variants in short reads

For the purpose of this study, we focused on insertions, deletions, duplications and inversions. A snakemake v7.25.0 (Mölder et al., 2021) pipeline was used to run smoove v0.2.8 (Pedersen et al., 2020) with default settings for SV calling of short‐read data, followed by genotyping and filtering. The pipeline was based on code published by (Bertolotti et al., 2020) and adapted for the data in this study. smoove is a wrapper that uses lumpy (Layer et al., 2014) to call SVs, duphold (Pedersen & Quinlan, 2019) to annotate SVs with information on fold‐change in flanking regions, and svtyper (Chiang et al., 2015) to genotype variants using discordant read information. The resulting vcf‐file was filtered with bcftools (Danecek et al., 2021) using the duphold threshold DHFFC (depth fold‐change relative to flanking regions) <0.7 for deletions and DHFFC >1.3 for duplications (thresholds investigated in (Pedersen & Quinlan, 2019)), and MSHQ (Mean Smoove Heterozygosity Score) ≥3 for all variants, as suggested by (Petersen et al., 2020). Only autosomal, biallelic variants were analysed.

### 2.3 Manual curation of structural variants

SVs were curated manually using samplot v1.3.0 (Belyeu et al., 2021) and plotcritic v1.0.1 (formerly known as svplaudit, (Belyeu et al., 2018)) to remove false positives. For each variant, SV calls from the three possible genotypes (homozygous reference, heterozygous, homozygous alternative allele) were plotted on top of each other (script was provided by Gabriel David and is available on github, see Data availability), with their overall coverage and discordant read pairs marked in different colours. All plots were scored using the plotcritic web interface. We chose to request two individuals of each genotype be available for manual inspection. This is a trade‐off between confidence and the ability to detect rare alleles: inspecting more individuals of each genotype is likely to lead to more confident calls, while requiring more individuals of each genotype will lead to a loss of rare variants (e.g., excluding singletons). In our data of 212 individuals, seeing at least two individuals homozygous for the alternative allele is expected for loci with a minor allele frequency (MAF) down to ≈0.01. However, many loci with a low MAF may not meet this criterion for stochastic reasons and because of population structure.

One curator scored all variants that met the above genotype criterion while the two smaller classes of SVs (duplications and inversions) were also independently scored also by a second curator, to assess whether additional manual curation could improve the quality of the data. By keeping only variants that both curators had labelled as correct, the retained set of variants was nearly half that of the set scored by one curator, without a notable effect on downstream analyses. We conclude that adding a second curator might over‐curate the data and chose to proceed with the set from a single curator. A comparison of a principal component analysis (see below) with kept variants using one or two curators is presented in Figure S1.

Some authors have defined SVs as variants at least 50 bp in length (Mahmoud et al., 2019). Since this is an arbitrary limited and likely has little biological meaning, we included all detected variants irrespective of length (very few were actually shorter than 50 bp). This should also be noted given that other authors have implemented a maximum length cut‐off, for example, at 50 kb (Wold et al., 2023).

### 2.4 Trio and population analysis as validation methods

Our dataset included four reproducing pairs with in total seven offspring, comprising seven family trios that could be used to assess the genotyping error rate. We compared the genotypes of each offspring to those of their parents and counted the fraction of variants that followed Mendelian inheritance. Sites with missing data for one or more individual(s) in each trio were excluded for this analysis.

Principal component analysis (PCA) was performed using plink v1.90b4.9 (Chang et al., 2015) with the flags ‘*‐‐dog ‐‐pca 20*’, generating the first 20 principal components for each SV type. We performed PCA on rejected and uncurated variants, and assessed potential batch effects (Figure S2). For rejected variants, and to some extent uncurated variants, samples from a particular sequencing batch separated from other samples in the PCA, which was not seen in the curated data. This indicates that samples sequenced together can share the same false variants, and we caution against technical biases in SV calling. The PCAs were plotted using R v4.2.1 (R Core Team, 2022) and the ggplot2 package v3.4.1 (Wickham, 2016).

### 2.5 Repeat analysis

Repeat annotation of the dog reference genome CanFam3.1 was downloaded from the UCSC genome browser and converted to bed format. Overlaps between variants and repeats were assessed with bedtools v2.29.2 (Quinlan & Hall, 2010) and classified using the repeat class name. A custom python script was used to ensure that in case of overlapping repeat annotations, each base was only counted once.

### 2.6 Overlap with genes, polarization and genomic load analysis

vep, Ensembl's Variant Effect Predictor (McLaren et al., 2016) is a common tool for considering the predicted effects of point mutations in gene sequences, assigning each codon an impact class (HIGH for nonsense mutations, MODERATE for non‐synonymous mutations, LOW for synonymous mutations, or MODIFIER for all other variants). Since SVs detected with our pipeline affect more than one codon (the shortest SV being a 14 bp deletion), we could not use vep or any similar tool for assessing deleteriousness. Instead, we divided SVs into three groups: those completely enclosing genes, those partially overlapping with coding sequence, and those only overlapping non‐coding parts of genes (for example, introns, UTRs). Each variant was assigned to only one category; a variant that completely enclosed one gene and partly overlapped with the other categories was assigned to the first category. For load analysis, we assigned the two first categories to be deleterious, and used variants in introns as a putatively neutral comparison. The rationale behind this was that SVs, which change the reading frame of proteins are highly likely to impair protein function. Even if the reading frame would not be changed, the fact that the vast majority of detected SVs was large (>50 bp) suggests that they would on average have negative effect on protein function.

For polarization of alleles we used the same two outgroups as in (Smeds & Ellegren, 2023) – African golden wolf and black‐backed jackal – and genotyped them for all identified SVs using the smoove wrapper. A variant was considered polarized only if both outgroups agreed and were homozygous for one of the alleles.

Genetic load was calculated as the fraction of putatively deleterious mutations in heterozygous state (masked load) or the fraction of deleterious mutations in homozygous state (realized load). All calculations and figures were made in R version 4.2.1 (R Core Team, 2022) using the vcfr (Knaus & Grünwald, 2017) and tidyverse (Wickham et al., 2019) packages.

## 3 RESULTS

### 3.1 Characterization of structural variants in the Fennoscandian wolf populations

Nearly 80,000 SVs (a total of 79,183, of which 62,706 were deletions, 7400 duplications and 9077 inversions) were identified and genotyped using short read data from 212 individuals. The variants were filtered for quality (with Duphold and MHSQ) and genotype frequency, and 35,807 variants (33,974 deletions, 2282 duplications and 550 inversions) with at least two individuals of each genotype were manually curated by visual inspection. After filtering and manual curation, 26,552 variants remained (25,640 deletions, 786 duplications and 126 inversions), removing 59.1%, 89.4% and 98.6% of the raw calls, respectively, for the different classes of variants (see Table 1). For duplications, the largest proportion was removed by the quality filtering step, while for deletions and inversions the largest proportions were removed due to the criterion of seeing at least two individuals of each possible genotype not being met, before manual curation. Of the variants remaining after filtering and manual curation, 23,116 segregated in the original Scandinavian population; the numbers were higher for Finnish and Russian wolves, 26,440 and 25,536 variants, respectively.

**TABLE 1:** Number of loci with structural variation in raw calls and after different filtering steps, separated by type of mutation.

Filtrering step	Deletions	Duplications	Inversions	Total
Raw calls	62,706	7400	9077	79,183
After duphold filtration	57,424	4372	6208	68,004
After genotype frequency filter^a^	32,975	2282	550	35,807
After manual curation	25,640	786	126	26,552

^a^ Loci with at least two individuals of each of the three possible genotypes.

**FIGURE 1:**
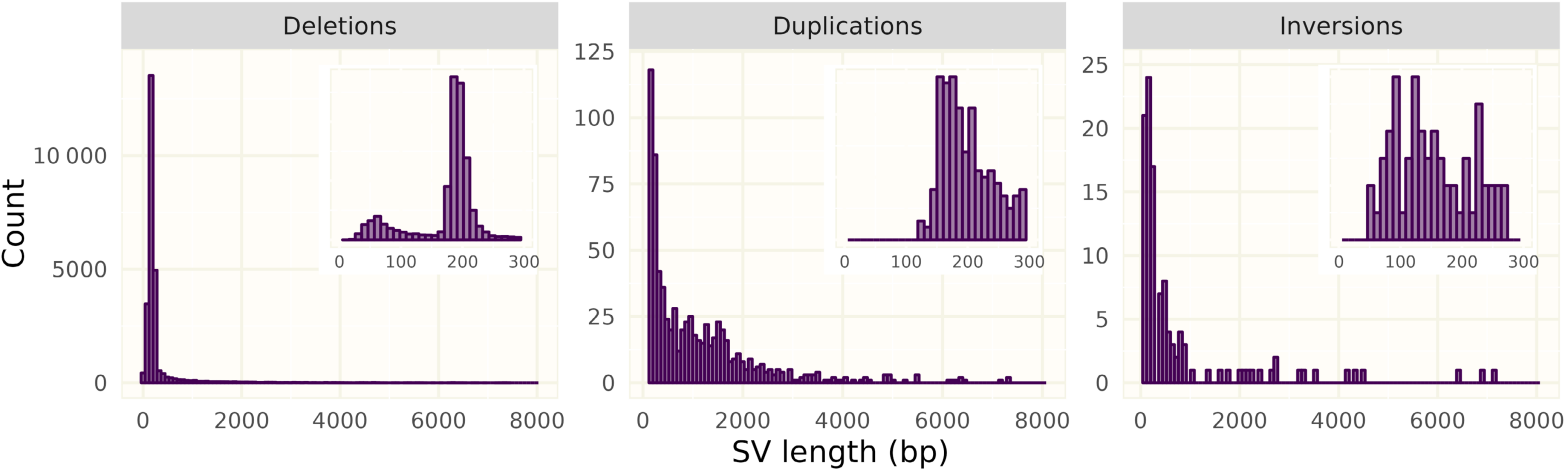
Length distributions for manually curated deletions, duplications and inversions. Insets show a close‐up for variants shorter than 300 bp. Variants longer than 7500 bp are not shown.

**FIGURE 2:**
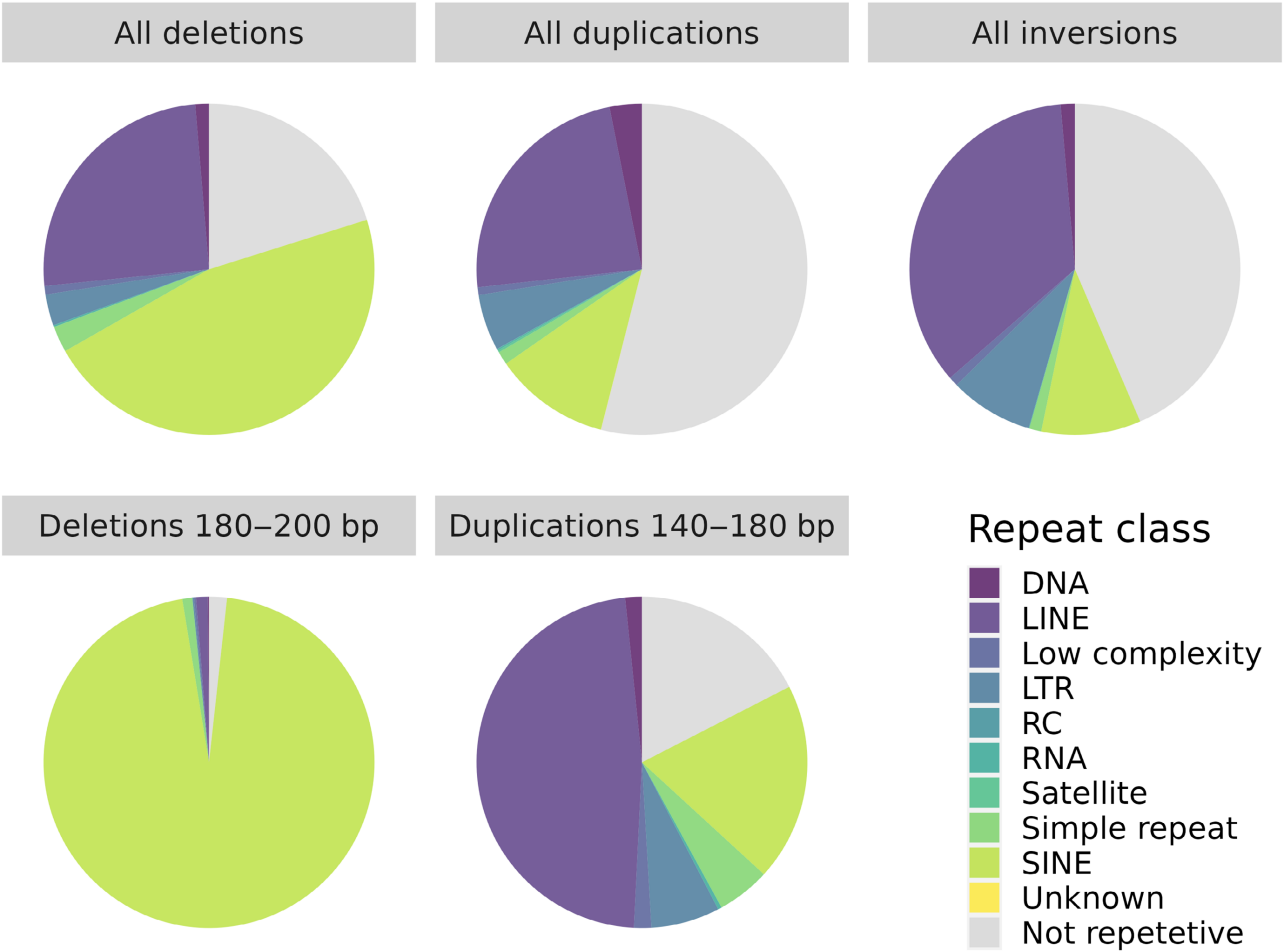
Repeat content of different classes of structural variants (top panels) along with the content for peaks in the length distributions of deletions at 180–200 bp and at 140–180 bp for duplications (bottom panels). Abbreviations for repeat classes: DNA, DNA repeat elements; LINE, Long interspersed nuclear elements; LTR, Long terminal repeat elements; RC, Rolling circle elements; RNA, RNA repeats; SINE, Short interspersed nuclear elements.

The vast majority of detected variants were longer than 100 bp (Figure 1; see also Figure S3) and variants shorter than 50 bp (Table S2) were found only among deletions. All three SV classes had variants exceeding 100 kb in length (a 112 kb deletion, a 442 kb duplication and a 170 kb inversion; see Figure S4 for samplot visualizations of these variants). Both deletions and duplications showed clear peaks at certain length classes: ≈190 bp for deletions and ≈160 bp for duplications. To test whether the peaks could be explained by frequent insertions of transposable elements (TE), we overlapped SVs with the repeat annotation of CanFam3.1 (Figure 2, top panel). The distinct 190 bp‐peak for deletions corresponded predominately to insertions of SINE/tRNA‐Lys elements. The 160 bp duplication peak showed an overrepresentation of LINE/L1 elements, though not as prominent as for the deletion peak (Figure 2, bottom panel).

### 3.2 Validation of structural variants

Most markers followed Mendelian inheritance: genotype concordance within seven parent‐offspring trios was high, around 95% (Table S3; see Figure S5, for example). Inversions had the highest fraction of variants that followed Mendelian inheritance, while deletions had the lowest. There were substantial differences in error rate among families and trios, with genotype concordance ranging from 92% to 99%. For the family with the highest incidence of mismatching genotypes (seen in two offspring trios), both parents were sequenced to a much lower coverage (<13×) than the rest of the samples (average 28.4×).

**FIGURE 3:**
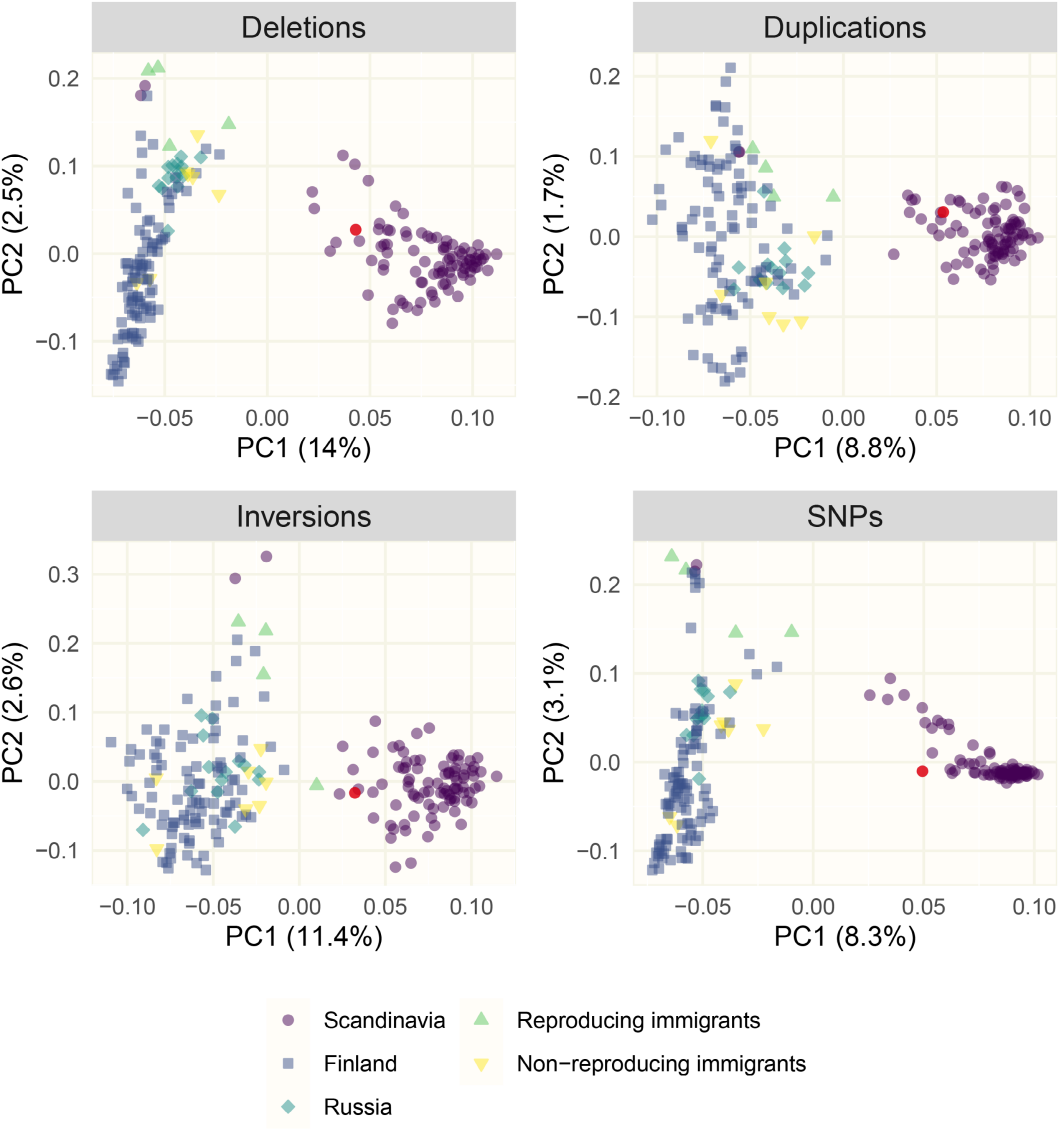
Population structure revealed by principal component analysis of 8,313,538 SNPs (from Smeds & Ellegren, 2023), 25,640 deletions, 786 duplications and 126 inversions. The duplication panel is mirrored for visualization purposes. “Scandinavia” (purple circles) includes all wolves born in Scandinavia, also offspring to reproducing immigrants. The female founder is highlighted in red.

As another means for validation, we assessed the population structure of Scandinavian, Finnish and Russian wolves using PCA of SV data (Figure 3). Immigrants to Scandinavia clustered with Finnish and Russian wolves, while most Scandinavian‐born wolves formed a separate cluster, as expected (see Section 4). Two Scandinavian‐born wolves clustered with Finnish/Russian individuals, which is not unexpected since they were first‐generation offspring to two immigrants (Kardos et al., 2018).

### 3.3 Structural variants in genes

Thirty‐eight deletions and 15 duplications completely enclosed 59 protein‐coding genes (Table 2). Furthermore, there were 83 variants (53 deletions, 29 duplications and 1 inversion) covering parts of coding sequences, in some cases including the start or stop codon. We consider the derived allele of variants involving coding sequence to be deleterious (~60% of the variants could be polarized, see Section 2). The polarization also allowed us to separate the category ‘deletions’ into deletion and insertion mutations. In our pipeline, deletions were identified in the short‐read data relative to the dog reference genome, which may either represent the ancestral state (and then the called deletion was a true deletion) or the derived state (in which case the called deletion was actually an insertion mutation).

**TABLE 2:** Number of loci with structural variation overlapping with protein‐coding genes, and the number of genes affected.

Type of overlap	No of loci with variants (genes affected)			
	Deletions	Duplications	Inversions	Sum
Enclosing full gene(s)	38 (38)	15 (21)	0 (0)	53 (59)
Partially overlapping	53 (53)	29 (36)	1 (1)	83 (90)
Non‐coding	11,873 (5989)	314 (306)	47 (44)	12,234 (6323)

*Note*: “Partially overlapping” are loci that span into parts of the coding sequence (CDS), and “Non‐coding” are loci only overlapping with non‐coding parts of genes (introns, UTRs).

There were about twice as many insertions than deletions, though this varied substantially among different length classes (Table S4). For variants between 100 and 200 bp, insertions represented 90% of the polarized variants owing to the 190 bp‐peak corresponding to SINE transposition events. In contrast, for the largest length classes (variants >10 kb), all polarized variants were actual deletions. For both duplications and inversions, most identified variants represented the derived state.

**FIGURE 4:**
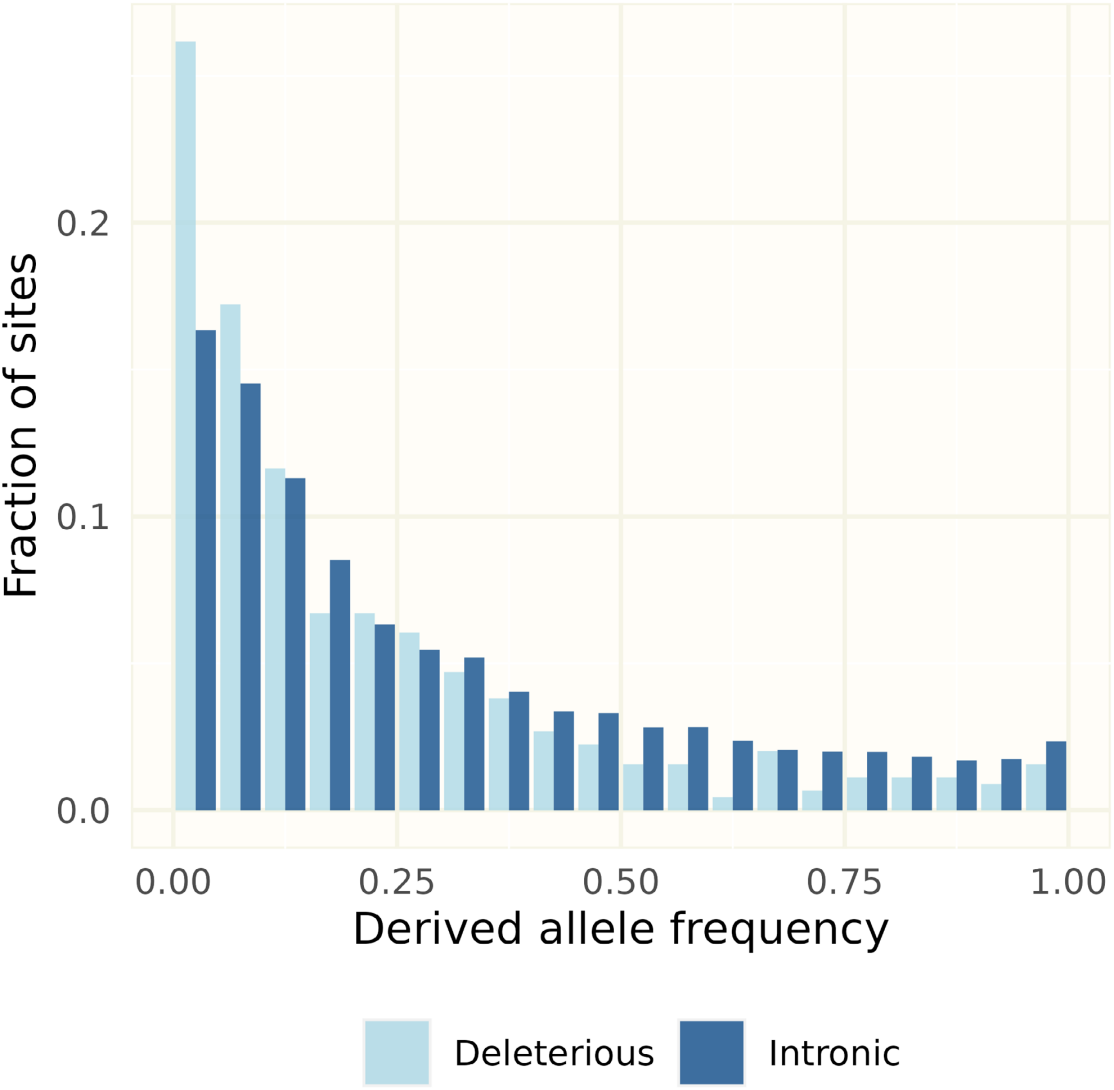
Allele frequency spectra of 40 unrelated wolves (17 Finnish, 13 Russian, 9 Scandinavian immigrants and the female founder), using deletions between 50 and 10,000 bp before the genotype frequency filter was applied, in order to keep rare variants. Derived allele frequencies are binned into 0.05‐intervals. Intronic variants are considered putatively neutral, deleterious are variants partly or fully overlapping with coding sequence.

To test whether SVs in protein‐coding genes are subject to purifying selection, we compared their unfolded allele frequency spectra with that of putatively neutral variants, here defined as variants in introns. However, since the genotype frequency filter effectively excluded all rare alleles, we used data before genotype frequency filtering for this analysis. This set is likely to contain a larger proportion of false positives, but by using deletions only from the 50–10,000 bp interval (the category and length classes with the highest fractions of variants remaining after manual curation, see Table S2) we sought to minimize noise from false variants. The allele frequency distribution for deletions that overlapped with coding sequences was significantly skewed to the left (Figure 4, Chi‐square goodness of fit test, *p* = 1.73e‐05) compared to putatively neutral variants, consistent with purifying selection.

Notably, there were a limited number of deletion variants in protein‐coding genes for which almost all individuals were homozygous for the derived allele, a pattern which initially suggested adaptive evolution. However, a closer inspection showed that the dog reference also carried the derived allele at these loci; thus, they were insertions present both in wolves and in the dog reference genome. This could be problematic for our analysis since genes were annotated based on the dog and sequences inserted in the ancestral lineage leading to wolves and dogs will be part of the annotation. We would erroneously interpret variants in these cases as deleterious (or adaptive) deletions. To avoid any bias caused by the annotation, we base our downstream genetic load analysis only on variants for which the dog reference represents the ancestral allele.

### 3.4 Genetic load and genetic rescue in the Scandinavian wolf population

**FIGURE 5:**
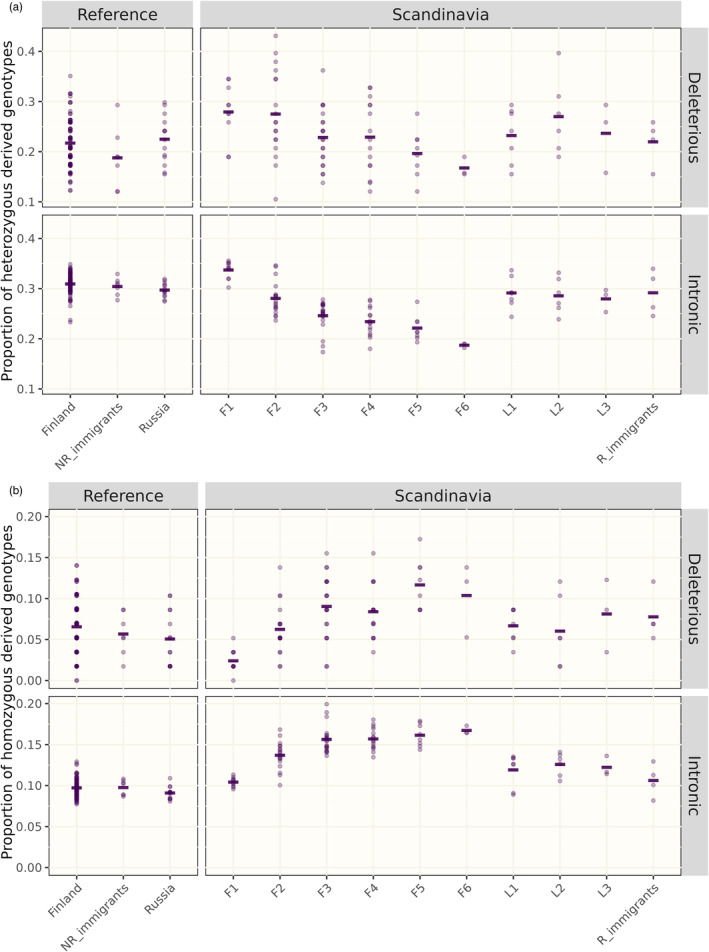
Proportion of (a) heterozygous genotypes (reflecting the masked load) and (b) homozygous derived genotypes (reflecting the realized load) for deleterious (overlapping with coding sequence, top panel) and intronic (putatively neutral, bottom panel) structural variants in different sets of wolf samples. Scandinavian‐born wolves are separated by number of generations to closest founder with descendants to the first three founders denoted F1–F6 and descendants to later reproducing immigrants denoted L1–L3. NR_immigrants, non‐reproducing immigrants; R_immigrants, reproducing immigrants. Mean values within each group are marked with horisontal bars.

The number of SVs in coding regions per population is shown in Table 3 (see also Table S5). Like for the total number of SVs, the numbers in the Russian and Finnish populations were higher than in the original Scandinavian population. We examined the effect of inbreeding and genetic drift in the original Scandinavian population on the occurrence of SVs in protein‐coding genes. After the founding event, the number of segregating SVs in genes – both in coding and in non‐coding sequences – decreased for every generation of inbreeding, indicative of a strong role of genetic drift (Table S6). We divided the genetic load into masked load (contributed by deleterious mutations in heterozygous state) and realized load (deleterious mutations in homozygous state) and found that the masked load decreased for every generation of inbreeding, while the realized load increased (Figure 5a,b, bottom panels). These observations are consistent with expectations from inbreeding, which shifts genotype frequencies towards increased homozygosity. Similar pattern was seen for intronic variants, with heterozygous genotypes decreasing and homozygous genotypes increasing with number of inbreeding generations (Figure 5a,b, top panels).

**TABLE 3:** Number of loci with polarized structural variants in protein‐coding genes per population, and mean ± SD per individual, only using loci where the dog reference had the ancestral allele.

Type of overlap	Original Scandinavia (*n* = 76)		Finland (*n* = 95)		Russia (*n* = 14)	
	No	Mean per individual	No	Mean per individual	No	Mean per individual
Enclosing full gene(s)	12	4 ± 2	17	5 ± 2	13	5 ± 2
Partially overlapping	28	15 ± 3	40	11 ± 2	34	11 ± 2
Non‐coding	1762	900 ± 58	2224	891 ± 32	2095	856 ± 30
Total	1802	919 ± 59	2281	907 ± 32	2142	872 ± 30

*Note*: Number of analysed individuals are denoted in parenthesis.

**FIGURE 6:**
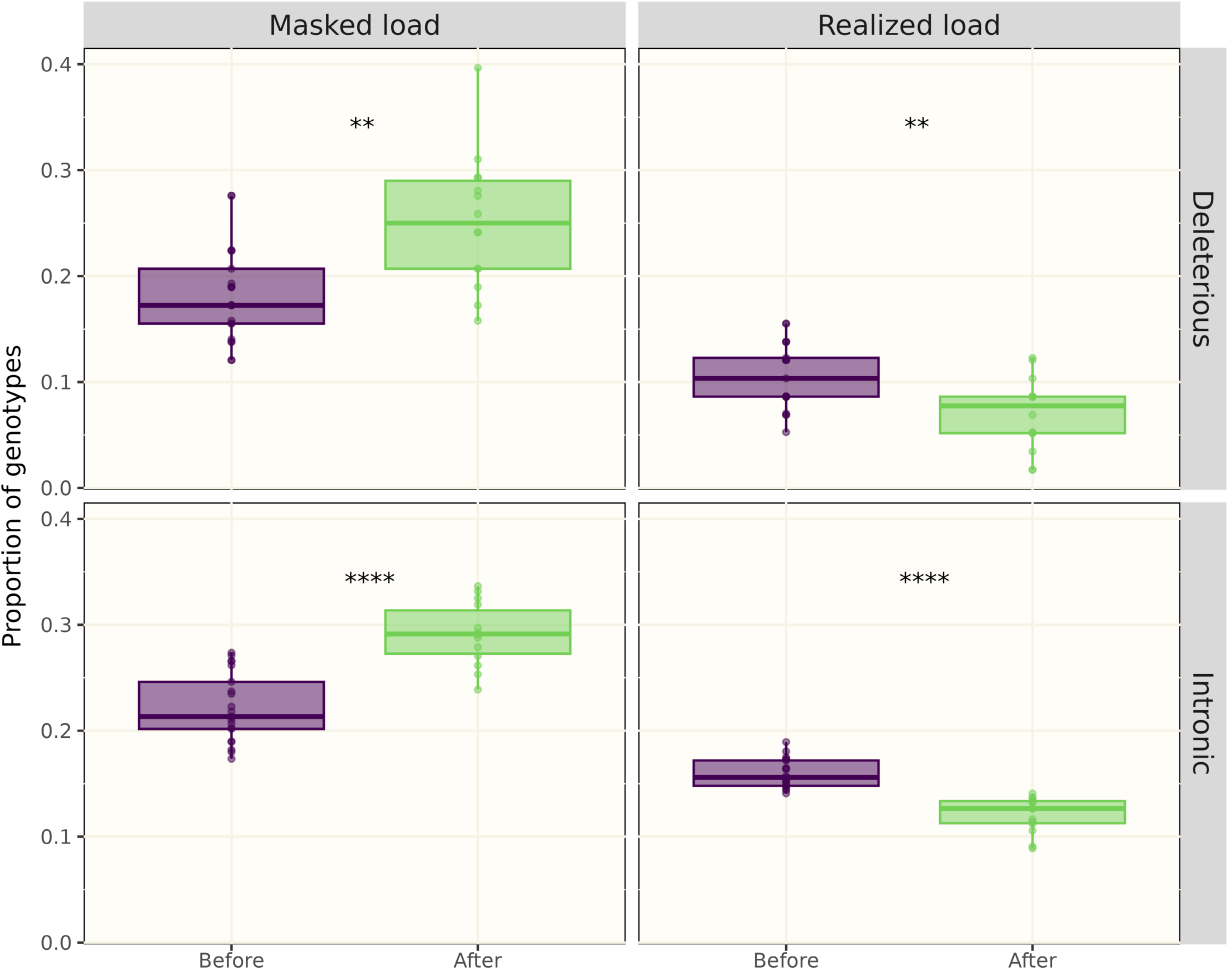
Box plots of the genetic load in the original Scandinavian wolf population before (purple) and after (green) immigration. The masked load are deleterious variants in heterozygous state (top left) and the realized load are homozygous deleterious variants (top right). The proportions of heterozygous and homozygous intronic variants (putatively neutral) are shown for comparison (bottom panel). **p < 0.01, ****p < 0.0001.

Next, we focused on the possible genetic rescue effect of the arrival of two immigrants in 2008 that bred in the population. The masked load was significantly higher in 14 immigrant offspring (that is, offspring from mating between an immigrant and an individual from the original population) compared to 21 descendants of the first three founders sampled during the same time period (that is, after several generations of inbreeding in the original population; Wilcoxon's test, *p* = 0.0013, Figure 6, bottom left). The realized load showed the opposite pattern, with lower levels in immigrant offspring (Wilcoxon's test, *p* = 0.0085, Figure 6, bottom right). These observations are consistent with outbreeding effects. Again, intronic variants showed a similar pattern with an increased proportion of heterozygotes and decreased proportion of homozygotes after immigration.

No SVs in coding regions went to fixation before immigration, but 45 non‐coding variants drifted to fixation for the derived allele after 5–6 generations of inbreeding. Of these, all but one variant regained the ancestral allele after immigration. Of 35 variants in the whole genome for which all three founders likely were homozygous for the derived allele (since no heterozygous genotypes were seen in the original population), all regained the ancestral allele after immigration. This demonstrates a strong genetic rescue effect even after limited immigration.

## 4 DISCUSSION

Characterization of genetic diversity has become an essential part of conservation biology and in studies of endangered species (Flanagan et al., 2018; Frankel & Soulé, 1981; Harrisson et al., 2014; Hohenlohe et al., 2021; Soulé, 1987; Theissinger et al., 2023). The field has seen a continuous development in terms of methodology, biological level (e.g., chromosomes, DNA, RNA, and protein), types of sequence diversity, and scale. In recent years, state‐of‐the‐art has been to analyse large‐scale sequence variation in the form of SNPs throughout the genome using data from whole‐genome short‐read sequencing. There has also been a trend to not only focus on neutral genetic diversity but also include analyses of functional diversity (Hoelzel et al., 2019), particularly so when it comes to adaptive and deleterious variants in coding sequences (or regulatory elements). Annotation of putatively deleterious mutations is necessary to estimate genetic load and to be able to study the distribution of fitness effects (Robinson et al., 2023); it is noteworthy that this can rarely be done and deleteriousness is only assumed.

The present study adds to a growing literature showing that another type of genetic diversity, structural variation, represents an abundant source of genetic diversity in natural populations (Chakraborty et al., 2019; Mérot et al., 2020), which has an impact on how endangered populations may respond to genetic drift and inbreeding (Wold et al., 2021). Moreover, using a recognized model in conservation biology, the grey wolf, we demonstrate that SVs in protein‐coding genes are subject to purifying selection and overall deleterious, that inbreeding increases the realized genetic load contributed by SVs, and that immigration provides a means for genetic rescue by re‐introducing ancestral alleles at SV loci. Before discussing these results, we highlight some methodological aspects of analysing structural genomic variation.

### 4.1 Methodological aspects

Our pipeline for identification and analysis of structural variation based on smoove, implementing lumpy‐sv for SV detection, found 79,183 autosomal variants in the raw calls of a sample of 212 wolves from three populations in northern Europe. The sample included more than 100 individuals from the highly inbred Scandinavian population and several related Finnish wolves, reducing the number of unrelated individuals to ≈40. After stringent quality and genotype frequency filtering, 35,807 variants remained, and manual curation further reduced the number to a final set of 26,552 variants. In comparison, a screen for SNPs using the same sequence data generated more than 10 million variants (Smeds & Ellegren, 2023), tentatively indicating that the number of SVs in the wolf genome is less than 1% that of SNPs.

Detection and genotyping of structural variation are technically more challenging than calling SNPs (Kosugi et al., 2019; Mahmoud et al., 2019; Wold et al., 2023). There can be large variation in the number and quality of SVs detected depending on a number of factors: the algorithm/s used for detection, procedures for filtering and curation, type of SVs, access to short‐ vs. long‐read data, quality of the reference genome, whether a pangenome is available, and the activity of transposable elements.

Even if the rate of false positives is typically higher in SV identification than in SNP analysis (Cameron et al., 2019; Ho et al., 2020; Mahmoud et al., 2019; see below), the number of SVs identified in our screen is likely to underestimate the actual number of variants present in the studied wolf genomes, and this is significantly so if one also considers very short indels. Our pipeline was neither optimized for detection of very short nor very long SVs. Of 1,153,466 short deletions identified with the common gatk pipeline for SNP detection (the vast majority representing one or two bp indels), only 819 were found in the present screen for structural variation. gatk identifies indels within single reads, while our pipeline identified SVs using read‐pair information and split reads. Conversely, of 883 deletions shorter than 50 bp found herein, around half (433) were called by gatk. On the other end of the length distribution of variants, the access to long‐read sequence data and data from optical mapping would have greatly facilitated detection of longer SVs (cf. (Audano et al., 2019; Chaisson et al., 2015; Sedlazeck et al., 2018), but see Zhao et al. (2021)).

Even when using multiple callers and conservatively taking the intersect of detected variants, false positives will remain (Wold et al., 2023). We chose instead to use a single general‐purpose caller but manually curated all variants by visual inspection. We requested at least two individuals of each genotype to be called and thus available for inspection to confidently judge a variant. An obvious drawback of this approach is that it excludes many rare variants from the accepted set of variants. To some extent, this was compensated by the relatively large number of genomes analysed (>200). Moreover, when estimating the site frequency spectrum of SVs we relaxed the selection criteria by also including variants for which less than two individuals were called homozygous for the rare allele. Possibly introducing noise, this is unlikely to affect the comparison of spectra for deleterious and neutral variants.

The access to several parent‐offspring trios offered a useful means for validation of the SV genotypes obtained in this study. About 95% of SV loci followed Mendelian expectations in all seven trios analysed, with a higher concordance rate of inversions and lower of duplications. This cannot directly be translated to genotyping error rate, but it gives an idea. There was a notable effect from sequence coverage: in two trios with (the same) parents sequenced to 12×, the concordance rate was 93.8%, while for five trios with a mean coverage of parent genomes of 28× the rate was 96.6%. This suggests that SV detection and genotyping using short‐read data should strive to use genomes sequenced at as high coverage as possible. Few studies are available to allow a direction comparison of observed rates of conformity with Mendelian inheritance. Data in Wold et al. (2023) (Table 3) from SV‐analyses of kākāpō (*Strigops habroptilus*) genomes indicates a somewhat higher incidence of violations of Mendelian inheritance than we observed, but the studies differ in design and methodology. Perhaps more importantly, pedigrees may often not be available in studies of natural populations. An alternative approach for validation of SV detection is to combine data from short‐ and long‐read sequencing. While large population samples may not, as yet, be possible to subject to long‐read sequencing, using a hybrid approach for a subset of individuals can reveal the error rate in short‐read data (Jiang et al., 2021; Wold et al., 2021).

An analysis of population structure among north European wolf populations using the curated set of SVs provided the same pattern as seen with SNPs (Smeds et al., 2021). Despite the Scandinavian population originally being founded by Finnish/Russian wolves, strong genetic drift in this small and isolated population has rendered allele frequencies different from those in the source population and hence separate the two populations in a PCA. Overall, these observations demonstrate that SVs can capture signals of population differentiation. Importantly, PCAs based on sets of SVs that included those not passing filtering or manual curation failed to detect the same pattern of population structure. This illustrates the necessity of stringent filtering and curation of SV raw data to allow making biologically meaningful conclusions.

### 4.2 Structural variants: genetic load and genetic rescue

Small populations are faced with the problem of losing genetic variation by random genetic drift, a process that may be a double‐edged sword: adaptive variants run the risk of being lost at the same time as deleterious variants risk drifting to fixation. Moreover, inbreeding, the likelihood of which increases with decreasing population size, will lead to the exposure of recessive deleterious alleles in homozygous form, resulting in inbreeding depression. While acknowledging that SVs may impact many genomic features and functions (e.g., regulatory elements, sequence modifications, chromatin structure and recombination landscape), we here solely focused on the effect of structural variation in protein‐coding genes.

The fact that the site‐frequency spectrum of SVs in coding sequences was significantly shifted towards rare alleles compared to SVs in putatively neutral sequences strongly indicates that, overall, structural changes in coding sequence are deleterious. This is not surprising and has been well documented for individual traits and loci in several species (see for example the cited supergene studies in the Introduction). It is also consistent with previous findings in species of conservation concern for deleterious SNPs in coding sequences, including in the Scandinavian wolf population (Smeds & Ellegren, 2023) as well as in alpine ibex (Grossen et al., 2020), Bengal tigers (Khan et al., 2021) and killer whales (Kardos et al., 2023). We thus concluded that SVs contribute to the genetic load of small populations and followed the segregation of SVs over time in the inbred Scandinavian wolf population. The frequency of homozygous variants – representing the realized genetic load – increased with the number of inbreeding generations, being more than twice as high after 5–6 generations.

Immigration clearly had positive effects on genetic diversity as manifested in patterns of structural variation. The arrival and integration of two eastern wolves into the Scandinavian population counteracted inbreeding, decreased the realized genetic load, and reintroduced ancestral alleles at loci that had become fixed for the derived allele in the population. These observations mirror what has been observed for deleterious point mutations in coding sequences in the Scandinavian wolf population (Smeds & Ellegren, 2023). Together, the joint effect of different types of mutations contribute to placing endangered populations in genetic peril, and call for the need of genetic rescue by gene flow – if possible – from neighboring populations or by translocations. In highly inbred populations, like the Scandinavian wolf population, regular immigration is necessary. In contrast, more sporadic gene flow is likely to only confer temporary positive effects for genetic diversity (cf. Lotsander et al. 2021).

Even if the number of SVs in a genome is far less than the number of SNPs, their sheer size may imply that they involve a larger proportion of the genome than point mutations. For example, Catanach et al. (2019) found that SVs accounted for diversity at three times the number of bases than SNPs in the marine teleost *Chrysophrys auratus*. In species with chromosome‐wide inversions, the relative excess of sites covered by SVs is likely to be even higher. Moreover, structural mutations in coding sequence often have more significant negative effects on protein function, i.e., being more deleterious, than point mutations (Conrad et al., 2010; Cridland et al., 2013; Emerson et al., 2008; Rogers, 2015). Association or genetic mapping studies in diverse organisms including fission yeast *Schizosaccharomyces pombe* (Jeffares et al., 2017), *Drosophila melanogaste*r (Chakraborty et al., 2019), American lobster *Homarus americanus* (Dorant et al., 2020), and the capelin *Mallotus villosus* (Cayuela et al., 2021) have revealed an overrepresentation of SVs in affecting important phenotypes. In fact, many structural mutations altering the reading frame or deleting sequences encoding functional sites/domains may be more or less lethal.

All variants called in this study were defined in relation to the dog reference genome. A variant called as a deletion could thus just as well be an insertion in the reference (and a called duplication could either be an insertion of a copy in our data or a deletion of a copy in the reference compared to the ancestral state). By using two outgroups for polarization, we could determine that the majority of ‘deletions’ in our set of wolves were in fact insertions of TE (SINE/tRNA‐Lys) elements in the dog reference genome. A particular family of these repeats, referred to as SINEC_Cf elements, is present in about 170,000 copies in the canine genome (Wang & Kirkness, 2005). They represent a very abundant source of presence‐absence polymorphisms in dog breeds, indicating their recent transposition activity and repeat expansion. Important to this study and to the genetic consequences of inbreeding in wolves, TE insertions have been shown to underlie a suite of genetic diseases and distinct phenotypes in dog breeds (Clark et al., 2006, 2008; Downs & Mellersh, 2014; Kehl et al., 2021; Marchant et al., 2017; Pelé et al., 2005). For example, a SINEC_Cf insertion in the *HCRTR2* gene causes narcolepsy in Doberman pinschers (Lin et al., 1999). The fact that the large majority of ‘deletions’ segregating in our sample of wolves represent TE insertions present in the dog reference genome implies that their origin predates dog domestication from wolves, their wild ancestor. It follows that wolves may be sensitive to inbreeding depression caused by recent TE insertions with similar phenotypic effects as in dogs.

## 5 CONCLUSIONS

This study demonstrates that structural variation represents a rich source of genetic diversity in wolf genomes. Many of these variants occur in protein‐coding sequences and are potentially deleterious; the fact that the allele frequency spectrum of such variants was significantly shifted towards rare alleles compared to putatively neutral variants, just as previously observed for potentially deleterious SNPs in coding sequence, supports this notion. Moreover, we conclude that potentially deleterious SVs have the same effect on genetic load as potentially deleterious SNPs: the masked load (proportion of heterozygotes) decreased with inbreeding but the realized load (proportion of homozygotes) increased. For both SVs and SNPs, immigration of wolves to the inbred Scandinavian wolf population decreased the realized load. These observations are consistent with expectations and demonstrate that the segregation of SVs follow the same population genetic processes as SNPs.

Large‐scale genotyping of SVs from whole‐genome sequence data is less straightforward and more prone to errors than conventional genotyping of SNPs. For a general characterization of levels and distribution of genetic diversity in natural populations, SNP genotyping thus compares favourably to genotyping of SVs. This is particularly so if long‐read sequencing is considered necessary for validation of SVs. However, conducting analytically challenging SV genotyping can be motivated as a complement to analyses of SNPs given that structural changes in protein‐coding sequences, and in other sequences subject to purifying selection (like regulatory elements), are likely to often have negative impact on fitness. A combined analysis of SVs and SNPs therefore provide a more complete picture of potentially deleterious variation.

Although the main focus of our study was to contribute to increased knowledge about structural genomic variation in endangered species, the findings presented herein can also be viewed in the more general context of the genomic consequences of inbreeding. Specifically, our results align with those of Mathur and DeWoody (2021) in showing an increase in realized genetic load with increased inbreeding. Other studies have suggested that purging of deleterious alleles can be sufficiently strong in small populations to reduce the realized load (Grossen et al., 2020). Since purging is a long‐term process, especially for mildly deleterious mutations, it may be too early to expect to see genome‐wide purging effects in the relatively recently founded Scandinavian wolf population.

## CONFLICT OF INTEREST STATEMENT

The authors report no conflict of interest.

## Supporting information


Appendix S1.

## Data Availability

The already published data used in this study are found in the European Nucleotide Archive (ENA), with accession numbers PRJEB20635, PRJEB28342, PRJEB39198 and PRJEB44869. Genotype data for the structural variants are available on Dryad (https://doi.org/10.5061/dryad.12jm63z57), and all scripts are available on github: https://github.com/linneas/wolf‐structural.
