# Supplementary material for: Structural genomic variation in the inbred Scandinavian wolf population contributes to the realized genetic load but is positively affected by immigration
Source: Evol Appl. 2024 Feb 7;17(2):e13652. doi: 10.1111/eva.13652 (PMC10848878; doi:10.1111/eva.13652)
Supplement: Supplementary file 1 — Appendix S1. [file EVA-17-e13652-s001.pdf]

Supplementary Materials

Structural genomic variation in the inbred Scandinavian wolf population contributes to the realized genetic load but is positively affected by immigration

Table S1. Meta data on all samples used in the study. Abbreviations: NR-immigrants = non-reproducing immigrants, R-immigrants = reproducing immigrants.

ID	Population	Generation Class	Sex	Seq Batch	X coverage	SRA_acc	Reference
D-00-12	Scandinavia	F3	female	2016	29	ERS1754868	Kardos et al. 2018
D-00-15	Scandinavia	F2	male	2016	33	ERS1754869	Kardos et al. 2018
D-01-18	Scandinavia	F2	male	2016	32	ERS1754870	Kardos et al. 2018
D-05-18	Scandinavia	NR-immigrants	male	2016	37	ERS1754871	Kardos et al. 2018
D-05-23	Scandinavia	F3	male	2016	35	ERS1754872	Kardos et al. 2018
D-06-14	Scandinavia	F3	female	2016	38	ERS1754873	Kardos et al. 2018
D-06-16	Scandinavia	F3	female	2016	35	ERS1754874	Kardos et al. 2018
D-07-09	Scandinavia	F5	male	2016	20	ERS1754875	Kardos et al. 2018
D-07-16	Scandinavia	F3	male	2016	23	ERS1754876	Kardos et al. 2018
D-07-17	Scandinavia	F5	female	2016	21	ERS1754877	Kardos et al. 2018
D-07-24	Scandinavia	F3	female	2016	14	ERS1754878	Kardos et al. 2018
D-07-28	Scandinavia	F3	male	2016	32	ERS1754879	Kardos et al. 2018
D-08-08	Scandinavia	F5	female	2016	24	ERS1754880	Kardos et al. 2018
D-08-10	Scandinavia	F3	female	2016	15	ERS1754881	Kardos et al. 2018
D-08-19	Scandinavia	F4	female	2016	30	ERS1754882	Kardos et al. 2018
D-08-20	Scandinavia	F4	male	2016	42	ERS1754883	Kardos et al. 2018
D-08-21	Scandinavia	F3	male	2016	16	ERS1754884	Kardos et al. 2018
D-10-15	Scandinavia	F6	female	2016	15	ERS1754885	Kardos et al. 2018
D-10-20	Scandinavia	F2	female	2016	31	ERS1754886	Kardos et al. 2018
D-10-23	Scandinavia	F6	female	2016	16	ERS1754887	Kardos et al. 2018
D-10-29	Scandinavia	F3	male	2016	25	ERS1754888	Kardos et al. 2018
D-10-30	Scandinavia	F3	male	2016	18	ERS1754889	Kardos et al. 2018
D-10-44	Scandinavia	F6	male	2016	25	ERS1754890	Kardos et al. 2018
D-10-50	Scandinavia	F4	male	2016	21	ERS1754891	Kardos et al. 2018
D-10-53	Scandinavia	L1	male	2016	20	ERS1754892	Kardos et al. 2018
D-10-68	Scandinavia	F4	male	2016	14	ERS1754893	Kardos et al. 2018
D-11-17	Scandinavia	F4	female	2016	29	ERS1754894	Kardos et al. 2018
D-11-19	Scandinavia	F2	male	2017	31	ERS6402637	Viluma et al. 2022

D-11-22	Scandinavia	F4	female	2016	11	ERS1754895	Kardos et al. 2018
D-11-58	Scandinavia	F4	female	2016	11	ERS1754896	Kardos et al. 2018
D-77-01	Scandinavia	NR-immigrants	male	2016	32	ERS1754897	Kardos et al. 2018
D-79-01	Scandinavia	NR-immigrants	female	2016	27	ERS1754898	Kardos et al. 2018
D-84-03	Scandinavia	F1	male	2016	46	ERS1754899	Kardos et al. 2018
D-85-01	Scandinavia	Founder	female	2016	40	ERS1754900	Kardos et al. 2018
D-85-02	Scandinavia	F1	male	2016	44	ERS1754901	Kardos et al. 2018
D-86-01	Scandinavia	F1	male	2016	34	ERS1754902	Kardos et al. 2018
D-89-01	Scandinavia	F2	male	2016	34	ERS1754903	Kardos et al. 2018
D-89-03	Scandinavia	F2	female	2016	35	ERS1754904	Kardos et al. 2018
D-91-01	Scandinavia	F2	female	2016	31	ERS1754905	Kardos et al. 2018
D-92-01	Scandinavia	F1	male	2016	36	ERS1754906	Kardos et al. 2018
D-92-02	Scandinavia	F1	male	2016	29	ERS1754907	Kardos et al. 2018
D-92-03	Scandinavia	F1	male	2017	38	ERS6402636	Viluma et al. 2022
D-92-05	Scandinavia	F2	male	2016	38	ERS1754908	Kardos et al. 2018
D-92-06	Scandinavia	F3	male	2016	39	ERS1754909	Kardos et al. 2018
D-93-01	Scandinavia	F1	male	2016	42	ERS1754910	Kardos et al. 2018
D-93-02	Scandinavia	F2	female	2016	35	ERS1754911	Kardos et al. 2018
D-93-03	Scandinavia	F2	female	2016	23	ERS1754912	Kardos et al. 2018
D-94-01	Scandinavia	F3	female	2016	27	ERS1754913	Kardos et al. 2018
D-96-01	Scandinavia	F1	male	2016	34	ERS1754914	Kardos et al. 2018
D-99-02	Scandinavia	F1	male	2016	35	ERS1754915	Kardos et al. 2018
G100-12	Scandinavia	L2	male	2016	13	ERS1754916	Kardos et al. 2018
G100-14	Scandinavia	L1	male	2016	37	ERS1754917	Kardos et al. 2018
G106-13	Scandinavia	L2	male	2016	35	ERS1754918	Kardos et al. 2018
G109-11	Scandinavia	L2	male	2016	13	ERS1754919	Kardos et al. 2018
G110-11	Scandinavia	F4	female	2016	31	ERS1754920	Kardos et al. 2018
G111-14	Scandinavia	L2	male	2016	20	ERS1754921	Kardos et al. 2018
G126-13	Scandinavia	F5	male	2016	12	ERS1754922	Kardos et al. 2018
G139-12	Scandinavia	L2	female	2016	11	ERS1754923	Kardos et al. 2018
G174-13	Scandinavia	F5	female	2016	38	ERS1754924	Kardos et al. 2018
G175-13	Scandinavia	F4	male	2016	40	ERS1754925	Kardos et al. 2018
G23-13	Scandinavia	R-immigrants	male	2016	13	ERS1754926	Kardos et al. 2018
G24-14	Scandinavia	F5	female	2016	9	ERS1754927	Kardos et al. 2018
G31-13	Scandinavia	R-immigrants	female	2016	13	ERS1754928	Kardos et al. 2018
G32-12	Scandinavia	F3	male	2016	24	ERS1754929	Kardos et al. 2018
G32-15	Scandinavia	L3	female	2016	39	ERS1754930	Kardos et al. 2018
G34-10	Scandinavia	F4	female	2016	33	ERS1754931	Kardos et al. 2018
G37-10	Scandinavia	L1	male	2016	13	ERS1754932	Kardos et al. 2018
G47-11	Scandinavia	L1	female	2016	36	ERS1754933	Kardos et al. 2018
G50-12	Scandinavia	F4	male	2016	12	ERS1754934	Kardos et al. 2018
G58-15	Scandinavia	F5	female	2016	24	ERS1754935	Kardos et al. 2018
G67-15	Scandinavia	L1	female	2016	24	ERS1754936	Kardos et al. 2018

G82-10	Scandinavia	NR-immigrants	female	2016	17	ERS1754937	Kardos et al. 2018
G87-12	Scandinavia	F5	female	2016	10	ERS1754938	Kardos et al. 2018
G9-05	Scandinavia	F2	male	2016	17	ERS1754939	Kardos et al. 2018
G97-13	Scandinavia	F3	male	2016	28	ERS1754940	Kardos et al. 2018
M-00-09	Scandinavia	F2	male	2016	46	ERS1754941	Kardos et al. 2018
M-00-10	Scandinavia	F3	male	2016	38	ERS1754942	Kardos et al. 2018
M-01-04	Scandinavia	F2	female	2016	15	ERS1754943	Kardos et al. 2018
M-01-06	Scandinavia	F3	female	2016	25	ERS1754944	Kardos et al. 2018
M-01-09	Scandinavia	F2	male	2017	42	ERS6402638	Viluma et al. 2022
M-01-10	Scandinavia	F3	female	2016	39	ERS1754945	Kardos et al. 2018
M-02-15	Scandinavia	NR-immigrants	male	2016	26	ERS1754946	Kardos et al. 2018
M-03-06	Scandinavia	F2	male	2016	42	ERS1754947	Kardos et al. 2018
M-03-07	Scandinavia	F3	female	2016	35	ERS1754948	Kardos et al. 2018
M-05-01	Scandinavia	NR-immigrants	male	2016	39	ERS1754949	Kardos et al. 2018
M-05-07	Scandinavia	F4	female	2016	42	ERS1754950	Kardos et al. 2018
M-06-03	Scandinavia	F4	male	2016	36	ERS1754951	Kardos et al. 2018
M-06-04	Scandinavia	F4	female	2016	25	ERS1754952	Kardos et al. 2018
M-07-02	Scandinavia	NR-immigrants	male	2016	34	ERS1754953	Kardos et al. 2018
M-07-06	Scandinavia	F4	male	2016	20	ERS1754954	Kardos et al. 2018
M-09-03	Scandinavia	R-immigrants	male	2016	32	ERS1754955	Kardos et al. 2018
M-09-05	Scandinavia	F3	male	2016	13	ERS1754956	Kardos et al. 2018
M-09-17	Scandinavia	F2	male	2016	13	ERS1754957	Kardos et al. 2018
M-10-04	Scandinavia	L1	female	2016	17	ERS1754958	Kardos et al. 2018
M-10-10	Scandinavia	R-immigrants	male	2016	37	ERS1754959	Kardos et al. 2018
M-11-02	Scandinavia	L1	female	2016	15	ERS1754960	Kardos et al. 2018
M-98-01	Scandinavia	F3	female	2016	36	ERS1754961	Kardos et al. 2018
M-98-02	Scandinavia	F2	male	2016	30	ERS1754962	Kardos et al. 2018
M-98-03	Scandinavia	F1	female	2016	29	ERS1754963	Kardos et al. 2018
M-98-08	Scandinavia	F2	male	2016	39	ERS1754964	Kardos et al. 2018
V113	Russia	-	male	2019	38	ERS4802900	Smeds et al. 2021
V114	Russia	-	male	2019	38	ERS4802901	Smeds et al. 2021
V115	Russia	-	male	2019	34	ERS4802902	Smeds et al. 2021
V116	Russia	-	female	2019	38	ERS4802903	Smeds et al. 2021
V117	Russia	-	female	2019	39	ERS4802904	Smeds et al. 2021
V119	Russia	-	female	2019	38	ERS4802905	Smeds et al. 2021
V120	Russia	-	female	2019	38	ERS4802906	Smeds et al. 2021
V126	Russia	-	male	2019	41	ERS4802907	Smeds et al. 2021
V132	Russia	-	male	2019	41	ERS4802908	Smeds et al. 2021
V134	Russia	-	female	2019	37	ERS4802909	Smeds et al. 2021
V136	Russia	-	female	2019	40	ERS4802910	Smeds et al. 2021
V141	Russia	-	female	2019	39	ERS4802912	Smeds et al. 2021
V142	Russia	-	male	2019	39	ERS4802913	Smeds et al. 2021
V143	Russia	-	female	2019	39	ERS4802914	Smeds et al. 2021

W10	Finland	-	male	2017	50	ERS2672862	Smeds et al. 2019
W100	Finland	-	male	2017	30	ERS2672863	Smeds et al. 2019
W101	Finland	-	male	2017	33	ERS2672864	Smeds et al. 2019
W11	Finland	-	male	2017	21	ERS2672865	Smeds et al. 2019
W12	Finland	-	male	2017	17	ERS2672866	Smeds et al. 2019
W13	Finland	-	male	2017	31	ERS2672867	Smeds et al. 2019
W14	Finland	-	male	2017	30	ERS2672868	Smeds et al. 2019
W15	Finland	-	male	2017	30	ERS2672869	Smeds et al. 2019
W16	Finland	-	female	2017	25	ERS4802877	Smeds et al. 2021
W17	Finland	-	male	2017	29	ERS2672870	Smeds et al. 2019
W18	Finland	-	female	2017	29	ERS4802878	Smeds et al. 2021
W19	Finland	-	male	2017	29	ERS2672871	Smeds et al. 2019
W20	Finland	-	male	2017	28	ERS2672872	Smeds et al. 2019
W21	Finland	-	male	2017	29	ERS2672873	Smeds et al. 2019
W22	Finland	-	male	2017	31	ERS2672874	Smeds et al. 2019
W23	Finland	-	female	2017	27	ERS4802879	Smeds et al. 2021
W24	Finland	-	male	2017	23	ERS2672875	Smeds et al. 2019
W25	Finland	-	male	2017	34	ERS2672876	Smeds et al. 2019
W26	Finland	-	male	2017	33	ERS2672877	Smeds et al. 2019
W27	Finland	-	male	2017	31	ERS2672878	Smeds et al. 2019
W28	Finland	-	female	2017	26	ERS4802880	Smeds et al. 2021
W29	Finland	-	female	2017	23	ERS4802881	Smeds et al. 2021
W3	Finland	-	female	2017	27	ERS4802882	Smeds et al. 2021
W30	Finland	-	female	2017	27	ERS4802883	Smeds et al. 2021
W31	Finland	-	male	2017	31	ERS2672879	Smeds et al. 2019
W32	Finland	-	female	2017	21	ERS4802884	Smeds et al. 2021
W33	Finland	-	female	2017	25	ERS4802885	Smeds et al. 2021
W34	Finland	-	male	2017	25	ERS2672880	Smeds et al. 2019
W35	Finland	-	male	2017	19	ERS2672881	Smeds et al. 2019
W36	Finland	-	male	2017	48	ERS2672882	Smeds et al. 2019
W37	Finland	-	female	2017	35	ERS4802886	Smeds et al. 2021
W38	Finland	-	male	2017	25	ERS2672883	Smeds et al. 2019
W4	Finland	-	female	2017	26	ERS4802887	Smeds et al. 2021
W40	Finland	-	female	2017	23	ERS4802888	Smeds et al. 2021
W41	Finland	-	male	2017	18	ERS2672884	Smeds et al. 2019
W42	Finland	-	female	2017	29	ERS4802889	Smeds et al. 2021
W43	Finland	-	female	2017	32	ERS4802890	Smeds et al. 2021
W44	Finland	-	male	2017	25	ERS2672885	Smeds et al. 2019
W45	Finland	-	female	2017	26	ERS4802891	Smeds et al. 2021
W46	Finland	-	male	2017	23	ERS2672886	Smeds et al. 2019
W47	Finland	-	female	2017	36	ERS4802892	Smeds et al. 2021
W48	Finland	-	female	2017	34	ERS4802893	Smeds et al. 2021
W49	Finland	-	female	2017	22	ERS4802894	Smeds et al. 2021

W5	Finland	-	male	2017	33	ERS2672887	Smeds et al. 2019
W50	Finland	-	male	2017	30	ERS2672888	Smeds et al. 2019
W51	Finland	-	male	2017	25	ERS2672889	Smeds et al. 2019
W52	Finland	-	male	2017	27	ERS2672890	Smeds et al. 2019
W53	Finland	-	male	2017	25	ERS2672891	Smeds et al. 2019
W54	Finland	-	female	2017	32	ERS4802895	Smeds et al. 2021
W55	Finland	-	female	2017	27	ERS4802896	Smeds et al. 2021
W56	Finland	-	female	2017	26	ERS4802897	Smeds et al. 2021
W57	Finland	-	male	2017	28	ERS2672892	Smeds et al. 2019
W58	Finland	-	male	2017	32	ERS2672893	Smeds et al. 2019
W59	Finland	-	male	2017	30	ERS2672894	Smeds et al. 2019
W6	Finland	-	male	2017	26	ERS2672895	Smeds et al. 2019
W60	Finland	-	male	2017	22	ERS2672896	Smeds et al. 2019
W61	Finland	-	female	2017	28	ERS4802898	Smeds et al. 2021
W62	Finland	-	female	2017	29	ERS4802899	Smeds et al. 2021
W63	Finland	-	male	2017	27	ERS2672897	Smeds et al. 2019
W64	Finland	-	male	2017	25	ERS2672898	Smeds et al. 2019
W65	Finland	-	male	2017	32	ERS2672899	Smeds et al. 2019
W66	Finland	-	male	2017	31	ERS2672900	Smeds et al. 2019
W67	Finland	-	male	2017	26	ERS2672901	Smeds et al. 2019
W68	Finland	-	male	2017	31	ERS2672902	Smeds et al. 2019
W69	Finland	-	male	2017	30	ERS2672903	Smeds et al. 2019
W7	Finland	-	male	2017	23	ERS2672904	Smeds et al. 2019
W70	Finland	-	male	2017	25	ERS2672905	Smeds et al. 2019
W71	Finland	-	male	2017	26	ERS2672906	Smeds et al. 2019
W72	Finland	-	male	2017	17	ERS2672907	Smeds et al. 2019
W73	Finland	-	male	2017	27	ERS2672908	Smeds et al. 2019
W74	Finland	-	male	2017	24	ERS2672909	Smeds et al. 2019
W75	Finland	-	male	2017	44	ERS2672910	Smeds et al. 2019
W76	Finland	-	male	2017	26	ERS2672911	Smeds et al. 2019
W77	Finland	-	male	2017	29	ERS2672912	Smeds et al. 2019
W78	Finland	-	male	2017	23	ERS2672913	Smeds et al. 2019
W79	Finland	-	male	2017	33	ERS2672914	Smeds et al. 2019
W8	Finland	-	male	2017	40	ERS2672915	Smeds et al. 2019
W80	Finland	-	male	2017	32	ERS2672916	Smeds et al. 2019
W81	Finland	-	male	2017	27	ERS2672917	Smeds et al. 2019
W82	Finland	-	male	2017	22	ERS2672918	Smeds et al. 2019
W83	Finland	-	male	2017	23	ERS2672919	Smeds et al. 2019
W84	Finland	-	male	2017	23	ERS2672920	Smeds et al. 2019
W85	Finland	-	male	2017	26	ERS2672921	Smeds et al. 2019
W86	Finland	-	male	2017	22	ERS2672922	Smeds et al. 2019
W87	Finland	-	male	2017	34	ERS2672923	Smeds et al. 2019
W88	Finland	-	male	2017	22	ERS2672924	Smeds et al. 2019

W89	Scandinavia	L3	male	2017	26	ERS2672925	Smeds et al. 2019
W9	Scandinavia	L2	male	2017	25	ERS2672926	Smeds et al. 2019
W90	Finland	-	male	2017	27	ERS2672927	Smeds et al. 2019
W91	Finland	-	male	2017	27	ERS2672928	Smeds et al. 2019
W92	Finland	-	male	2017	23	ERS2672929	Smeds et al. 2019
W93	Finland	-	male	2017	28	ERS2672930	Smeds et al. 2019
W94	Finland	-	male	2017	25	ERS2672931	Smeds et al. 2019
W95	Finland	-	male	2017	26	ERS2672932	Smeds et al. 2019
W96	Finland	-	male	2017	24	ERS2672933	Smeds et al. 2019
W97	Finland	-	male	2017	40	ERS2672934	Smeds et al. 2019
W98	Finland	-	male	2017	20	ERS2672935	Smeds et al. 2019
W99	Scandinavia	L3	male	2017	33	ERS2672936	Smeds et al. 2019

Table S2. Number loci with structural variation per length class in raw calls and after the different filtering steps.

SV Type	SV Length	1-50bp	51-100bp	101-200bp	201-300bp	301-400bp	401-500bp	501bp-1kb	1kb-10kb	10kb-50kb	>50kb	Sum
Deletions	**Raw calls**	4,085	5,515	23,162	12,126	2,689	1,862	4,138	5,962	836	2,331	62,706
	**After duphold filtration**	3,584	5,072	22,841	11,912	2,482	1,600	3,545	4,829	369	1,190	57,424
	**After genotype filter**	2,588	3,217	14,855	6,527	1,117	743	1,649	2,112	50	117	32,975
	**After manual curation**	883	2,574	13,569	5,513	592	382	941	1157	28	1	25,640
Duplications	**Raw calls**	0	3	554	552	331	237	834	1,408	682	2,799	7,400
	**After duphold filtration**	0	2	522	493	240	136	580	1,020	200	1,179	4,372
	**After genotype filter**	0	2	250	198	132	97	347	622	107	527	2,282
	**After manual curation**	0	0	123	92	51	32	136	313	31	8	786
Inversions	**Raw calls**	78	127	1,409	723	410	491	2,434	2,796	204	405	9,077
	**After duphold filtration**	74	111	1,110	556	291	361	1,334	2,047	118	206	6,208
	**After genotype filter**	39	40	60	76	44	35	88	134	18	16	550
	**After manual curation**	0	16	29	17	4	10	17	23	9	1	126

*All loci with at least two individuals of each of the three possible genotypes.

Table S3. Structural variant genotyping accuracy based on parent-offspring trios.

**Group**	**Family**	**Parents (X coverage)**	**Offspring (X coverage)**	**Percentage variants following Mendelian inheritance**		
				**Deletions**	**Duplications**	**Inversions**
**Immigrants**	1	Mother (12.7X), Father (12.8X)	Offspring 1 (37.3X)	92.8%	93.0%	96.0%
			Offspring 2 (24.3 X)	92.8%	91.7%	96.8%
**Scandinavia**	2	Mother (28.9X), Father (30.4X)	Offspring 1 (38.9X)	99.4%	95.2%	98.4%
			Offspring 2 (42.3X)	93.9%	94.1%	98.4%
**Finland**	3	Mother (26.2X), Father (28.1X)	Offspring 1 (31.7X)	94.8%	95.0%	98.4%
**Finland**	4	Mother (31.8X), Father (24.7X)	Offspring 1 (21.8X)	98.8%	91.8%	99.2%
			Offspring 2 (29.3X)	99.1%	94.0%	99.2%
**Average**				95.9%	93.6%	98.1%

Table S4. Number of loci with polarized structural variants per length class, and numbers of loci where the dog reference carries the ancestral allele (“Ancestral is REF”) or the derived allele (“Ancestral is ALT”), respectively.

SV Type	SV Length	1-50bp	51-100bp	101-200bp	201-300bp	301-400bp	401-500bp	501-1kb	1kb-10kb	10-50kb	>50kb	Sum
**Deletions**	After manual curation	883	2,574	13,569	5,513	592	382	941	1,157	28	1	25,640
	Polarized, in total	534	1,785	8,291	2,377	418	270	692	920	24	1	15,312
	Ancestral is REF	170	1,278	970	419	238	176	495	813	24	1	4,584
	Ancestral is ALT	364	507	7,321	1,958	180	94	197	107	0	0	10,728
**Duplications**	After manual curation	0	0	123	92	51	32	136	313	31	8	786
	Polarized, in total	0	0	54	28	10	6	97	272	27	7	501
	Ancestral is REF	0	0	53	26	10	6	96	268	28	7	494
	Ancestral is ALT	0	0	1	2	0	0	1	4	1	0	9
**Inversions**	After manual curation	0	16	29	17	4	10	17	23	9	1	126
	Polarized, in total	0	12	17	5	2	5	10	18	7	1	77
	Ancestral is REF	0	7	14	5	2	5	8	14	7	1	63
	Ancestral is ALT	0	5	3	0	0	0	2	4	0	0	14

Table S5. Number of loci with structural variation in protein-coding genes seen per population and mean per individual (± SD). AA=REF denotes that the dog reference has the ancestral allele, and AA=ALT that the dog reference has the derived allele.

SV Type		Original Scandinavia (n=76) No	Original Scandinavia (n=76) Mean per individual	Finland (n = 95) No	Finland (n = 95) Mean per individual	Russia (n = 14) No	Russia (n = 14) Mean per individual
Deletions	Enclosing full gene(s), AA=REF	5	2 ± 1	7	3 ± 1	6	3 ± 1
	Enclosing full gene(s), AA=ALT	16	12 ± 1	20	13 ± 2	20	13 ± 2
	Partially overlapping coding variants*, AA=REF	13	7 ± 2	16	5 ± 1	15	6 ± 2
	Partially overlapping coding variants*, AA=ALT	17	11 ± 2	18	12 ± 2	18	11 ± 1
	Non-coding variants**, AA=REF	1,602	827 ± 53	2,011	816 ± 29	1,910	787 ± 32
	Non-coding variants**, AA=ALT	5,002	3,205 ± 320	5,082	3,831 ± 276	5,007	3,170 ± 27
Duplications	Enclosing full gene(s), AA=REF	7	2 ± 1	10	2 ± 1	7	2 ± 1
	Enclosing full gene(s), AA=ALT	1	1 ± 0	1	1 ± 0	1	1 ± 0
	Partially overlapping coding variants*, AA=REF	15	8 ± 2	24	6 ± 2	19	6 ± 1
	Partially overlapping coding variants*, AA=ALT	0	NA	0	NA	0	NA
	Non-coding variants**, AA=REF	137	62 ± 6	187	63 ± 6	160	57 ± 7
	Non-coding variants**, AA=ALT	4	4 ± 1	4	4 ± 1	4	4 ± 0
Inversions	Enclosing full gene(s), AA=REF	0	NA	0	NA	0	NA
	Enclosing full gene(s), AA=ALT	0	NA	0	NA	0	NA
	Partially overlapping coding variants*, AA=REF	0	NA	0	NA	0	NA
	Partially overlapping coding variants*, AA=ALT	1	1 ± 0	1	1 ± 0	1	1 ± 0
	Non-coding variants**, AA=REF	23	11 ± 2	26	12 ± 2	25	12 ± 2
	Non-coding variants**, AA=ALT	3	2 ± 1	3	2 ± 1	3	1 ± 0
All	Total, AA=REF	1,802	919 ± 59	2281	907 ± 32	2,142	872 ± 30
	Total, AA=ALT	5,044	3,235 ± 322	5129	3,863 ± 276	5,054	3,202 ± 29

*Loci overlapping with CDS and/or start or stop codon.

**Loci overlapping with non-coding parts of genes (UTRs and introns).

Table S6. Number of loci with structural variation in protein-coding genes seen in samples of wolves divided according the number of generations of inbreeding since founding of the population. Only including loci for which the dog reference has the ancestral allele. The number of sampled individuals for each generation is denoted in parenthesis.

	**Generations to founders**					
	**1 (n=10)**	**2 (n=18)**	**3 (n=21)**	**4 (n=15)**	**5 (n=8)**	**6 (n=3)**
**Enclosing full gene(s)**	11	11	10	10	7	5
**Partially overlapping**	28	28	28	28	26	16
**Non-coding**	1,711	1,675	1,648	1,597	1,491	1,018

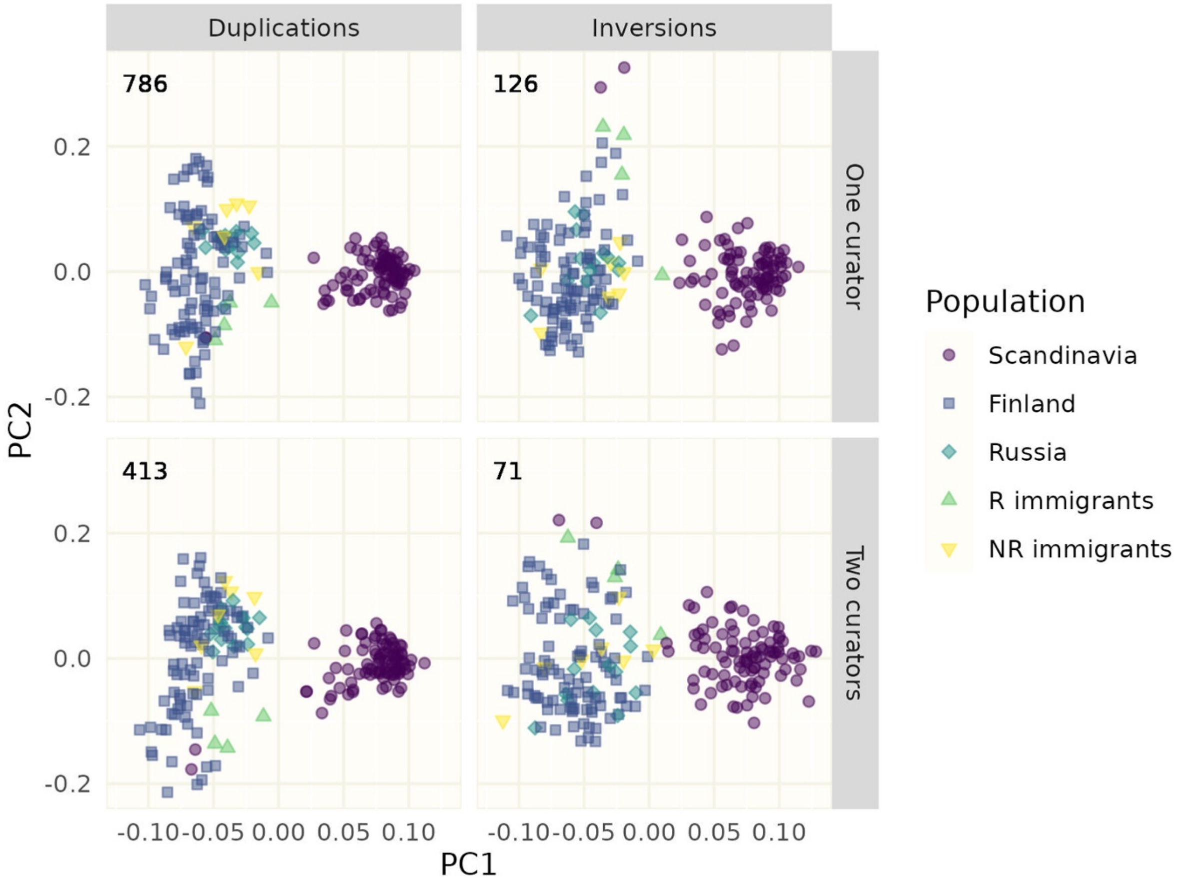

Figure S1: Principal component analysis of duplications and inversions using one (top panel) or two curators (bottom panel). Numbers of markers used for each PCA are denoted in the top left corners.

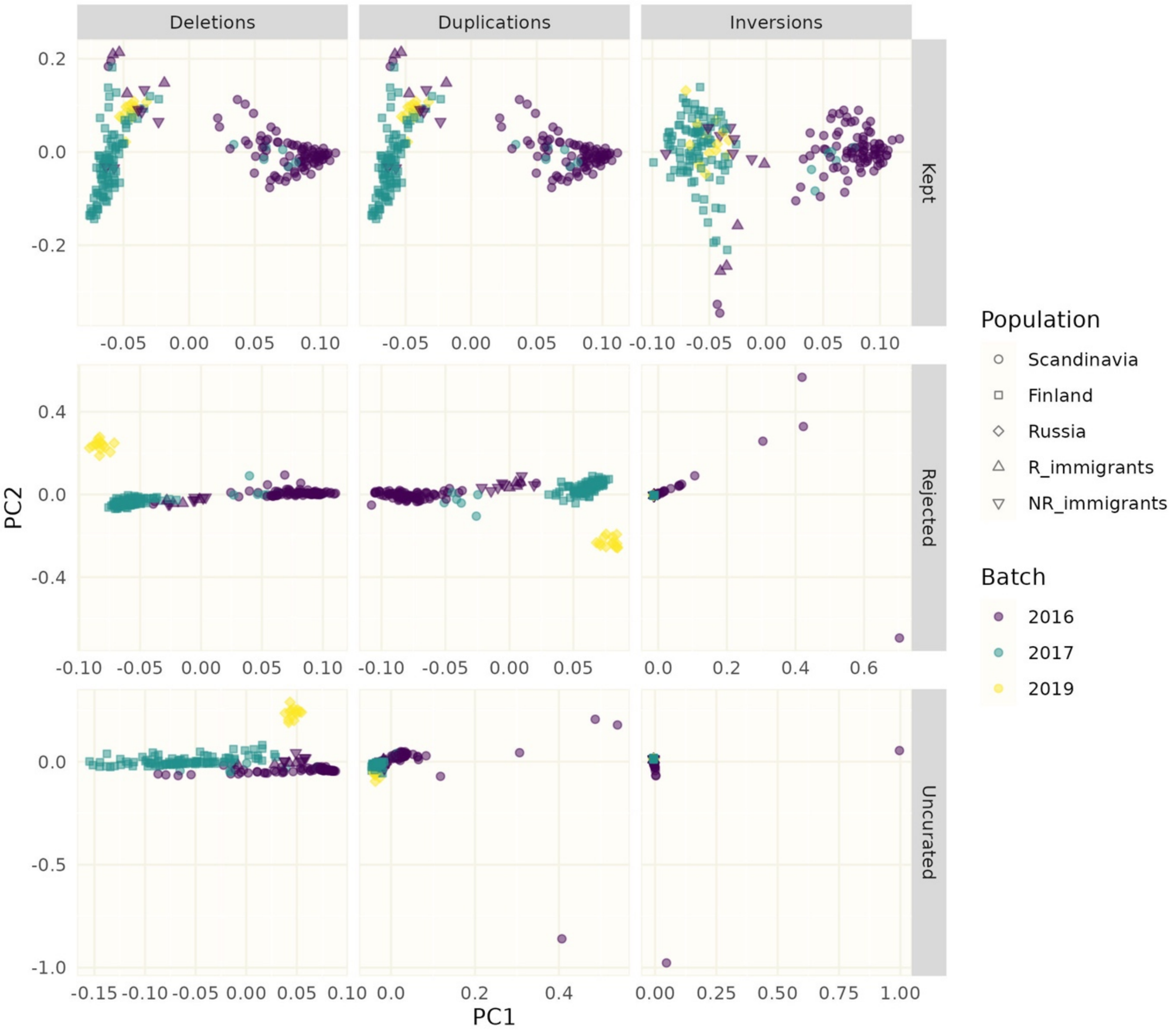

Figure S2. Principal component analysis of kept (upper panel), rejected (middle; either from duphold filtering or manual curation) and uncurated variants (lower; not passing genotype frequency filter). Symbols denote populations (R_immigrants = reproducing immigrants, NR_immigrants = non-reproducing immigrants) and colours which batch a sample was sequenced in. There is a batch effect especially for the 2019 batch among rejected and uncurated variants that is not seen among the kept variants.

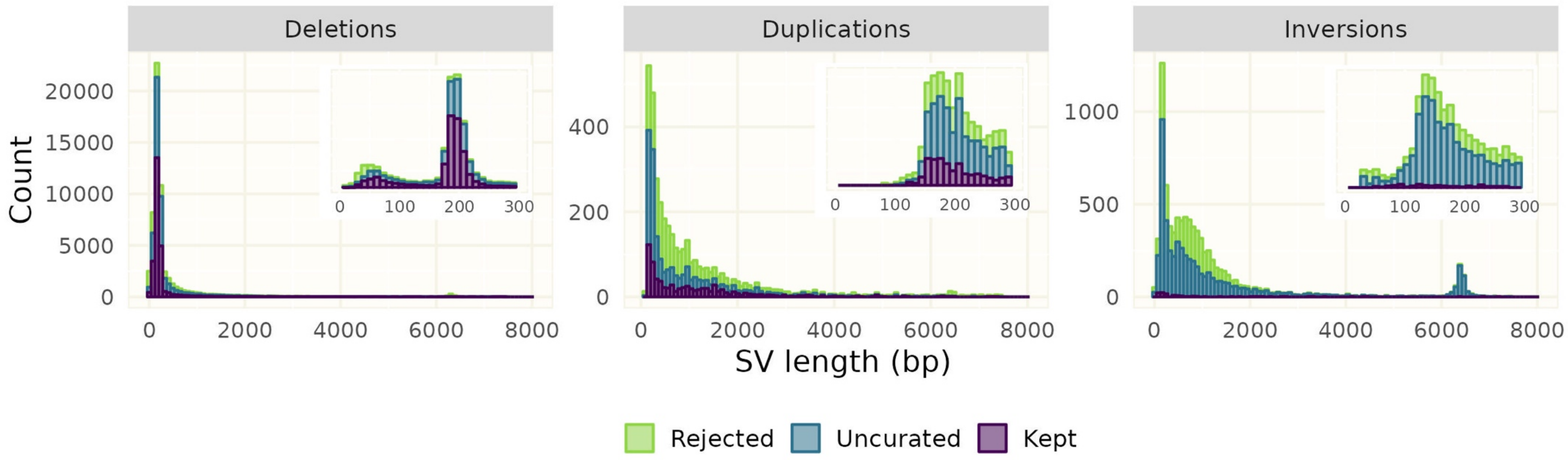

Figure S3. Length distributions of the different types of structural variants kept after filtering (Kept), variants that were rejected (Rejected; from duphold filter or manual curation), and variants not passing genotype frequency filter (Uncurated). Variants longer than 7,500bp are not shown. Insets show close-up of variants shorter than 300bp.

(a)

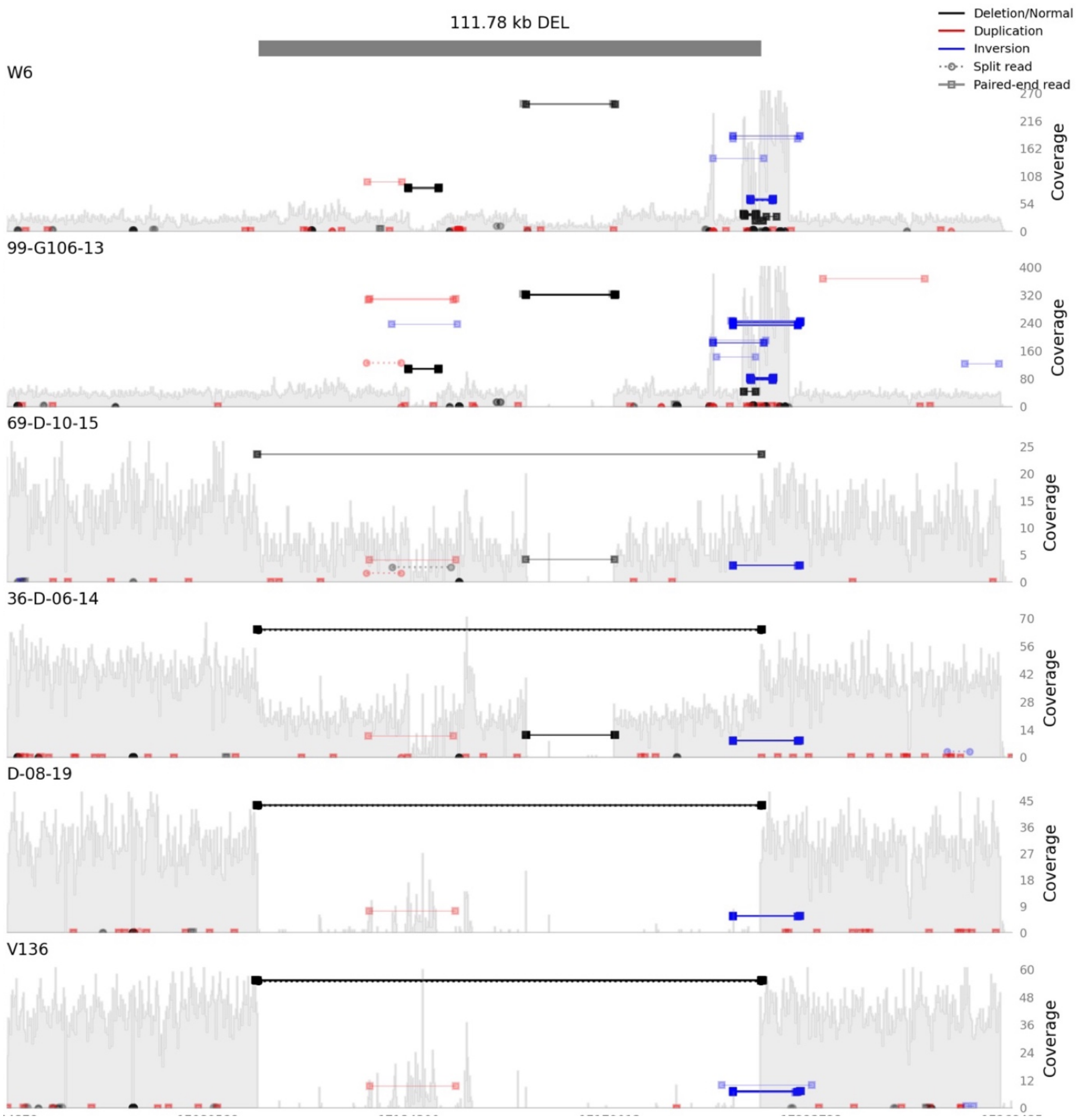

111.78 kb DEL
Deletion/Normal
Duplication
Inversion
Split read
Paired-end read
W6
99-G106-13
69-D-10-15
36-D-06-14
D-08-19
V136
Coverage

(b)

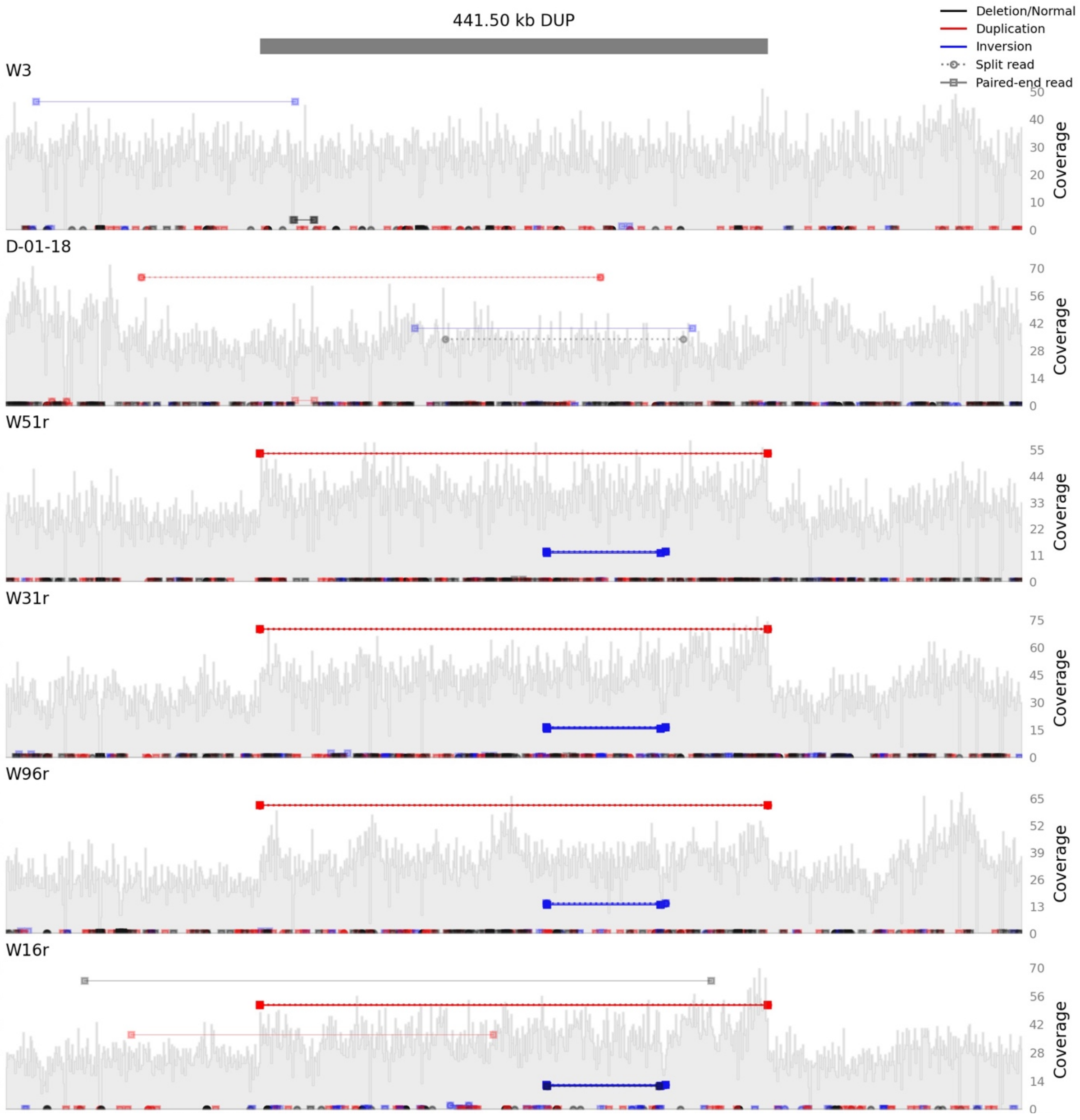
441.50 kb DUP
Deletion/Normal
Duplication
Inversion
Split read
Paired-end read
W3
D-01-18
W51r
W31r
W96r
W16r
Coverage

(c)

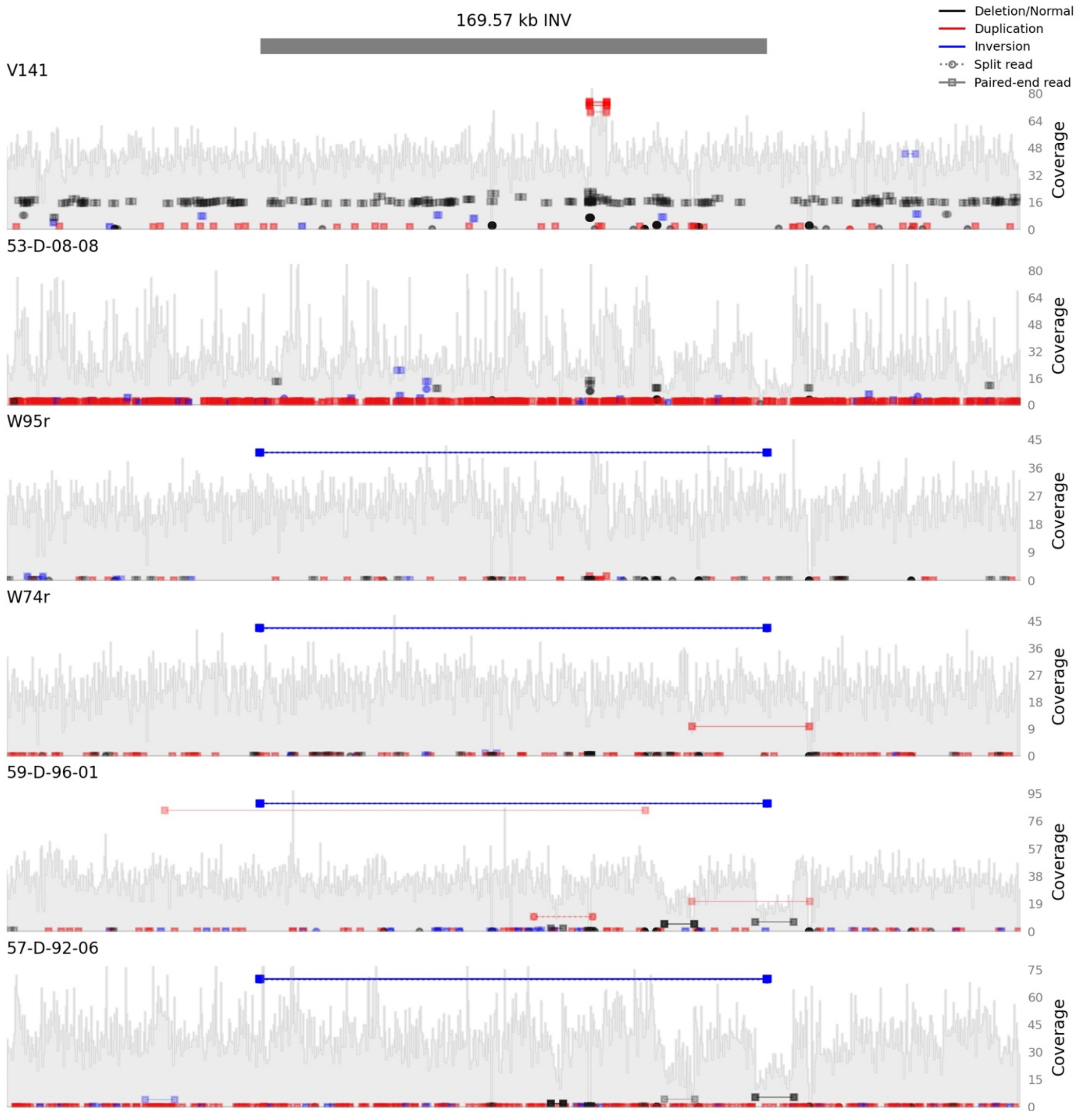

Figure S4. SAMPLOT visualization of the largest structural variants of each type: (a) a 112 kb deletion on chromosome 15, (b) a 442 kb duplication on chromosome 7, and (c) a 170 kb inversion on chromosome 21. For each loci, two individuals per genotype were randomly chosen for display with homozygous for reference allele shown on top, heterozygous in the middle, and homozygous for alternative allele in the bottom. The coloured lines represent read pairs supporting different type of variants (black = deletion, red = duplication, blue = inversion). For the deletions and duplications, evidence also comes from coverage information (in grey).

(a)

(b)

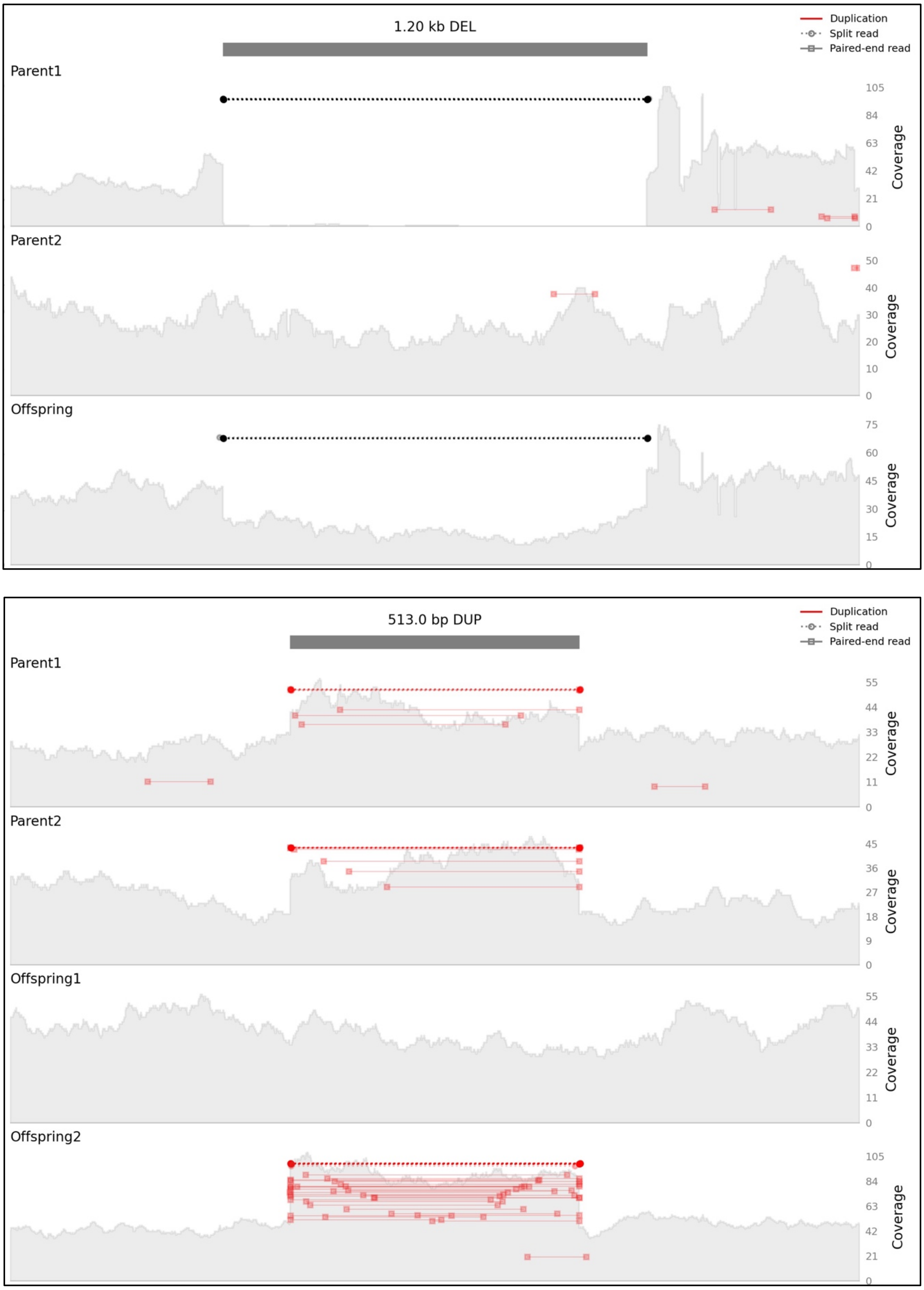

Figure S5. SAMPLOT examples of segregation of structural variants in family trios. (a) a 1.2 kb deletion with one parent homozygous for the reference allele and the other homozygous for the alternative allele, with a heterozygous offspring. (b) A 513 bp duplication with two heterozygous parents, with two offspring homozygous for the respective alleles.
